# Pseudorabies virus DNA polymerase processivity factor pUL42 inhibits type I IFN production by negatively regulating cGAS-STING signaling pathway

**DOI:** 10.1128/jvi.01218-25

**Published:** 2025-09-30

**Authors:** Guangqiang Ye, Jiaxiu Gao, Haoxuan Cao, Xiaohong Liu, Hongyang Liu, Shanghui Wang, Yunfei Liu, Longfei Han, Qiongqiong Zhou, Yandong Tang, Jin Tian, Liping Huang, Li Huang, Zhaoxia Zhang, Changjiang Weng

**Affiliations:** 1 Division of Fundamental Immunology, National African Swine Fever Para-reference Laboratory, State Key Laboratory for Animal Disease Control and Prevention, Harbin Veterinary Research Institute, Chinese Academy of Agricultural Sciences 111613 , Harbin, China; 2 Key Laboratory of Zoonosis Prevention and Control of Guangdong Province, College of Veterinary Medicine, South China Agricultural University 162683, Guangzhou, Guangdong, China; 3 Heilongjiang Provincial Key Laboratory of Veterinary Immunology, Harbin, China; University of Toronto, Toronto, Ontario, Canada

**Keywords:** pseudorabies virus, DNA polymerase processivity factor, pUL42, cGAS-STING, interferon

## Abstract

Pseudorabies (PR), caused by the PR virus (PRV), is an acute infectious disease in livestock and various wild animals. PRV has developed several immune evasion mechanisms to antagonize the host’s antiviral immune response. However, the precise role of PRV-encoded proteins in regulating the cyclic GMP-AMP synthase (cGAS)-stimulator of interferon (IFN) gene (STING) signal for immune evasion remains unclear. In this study, we demonstrate that the PRV UL42 protein (pUL42) inhibited cGAS-mediated antiviral signaling by modulating cGAS recognition of dsDNA. Mechanistically, pUL42 interacts with the DNA-binding domain of cGAS, thereby inhibiting its recognition of double-stranded DNA, leading to the inhibition of its dimerization and oligomerization activation. Furthermore, knocking down the expression of the *Ul42* gene in the PRV genome diminishes the antagonistic effect on type I IFN production and inhibits PRV replication. Ultimately, we have established that pUL42 targets cGAS-mediated signaling, thereby inhibiting the production of type I IFN and facilitating viral replication. Overall, our findings suggest that PRV pUL42 functions as an antagonist to evade the host’s antiviral response by targeting the cGAS-STING axis.

**IMPORTANCE:**

Cyclic GMP-AMP synthase (cGAS)-stimulator of interferon gene (STING) axis is essential for host resistance to DNA virus infections by regulating type I interferon production. However, whether pseudorabies virus (PRV) antagonizes the cGAS-STING signaling pathway to immune evasion is not fully investigated. In this study, we clearly demonstrated that the PRV pUL42 protein inhibits the recognition of double-stranded DNA of cGAS, leading to inhibiting the oligomerization and activation of cGAS, thereby suppressing the cGAS-mediated host antiviral immune responses. Taken together, our results reveal a novel strategy employed by PRV to evade host defenses, which will provide theoretical support for the development of anti-PRV drugs for the prevention and control of PRV.

## INTRODUCTION

The innate immune system is the host’s first line of defense against invading pathogens. Upon the invasion of pathogenic microorganisms, pattern recognition receptors (PRRs) in host cells detect pathogen-associated molecular patterns (PAMPs) and rapidly initiate a series of signaling events. This cascade ultimately leads to the production of type I interferon (IFN) and the expression of hundreds of other IFN-induced genes (ISGs) (1). The cyclic GMP-AMP synthase (cGAS) has been identified as a universal sensor for recognizing double-stranded DNA (dsDNA) in intracellular environments during DNA viral infection (2). cGAS detects dsDNA to induce the synthesis of the second messenger cyclic GMP-AMP (cGAMP), which then binds to and activates the stimulator of IFN gene (STING) factor, resulting in facilitating its translocation from the endoplasmic reticulum to the Golgi apparatus (3). Subsequently, STING recruits TANK-binding kinase (TBK1) and activates its phosphorylation (4). The active TBK1 phosphorylates the transcription factor IFN regulatory factor (IRF3), leading to its entering the nucleus and initiating type I IFN production (5).

Pseudorabies virus (PRV), one of the members of the alpha herpesvirus subfamily, is the pathogen of Aujeszky’s disease, which also threatens most mammals, including domestic pigs and wild boars (6, 7). PRV primarily invades the peripheral nervous system first and causes severe clinical and neurological symptoms, which can lead to the acute death of piglets (8). Although PRVs have not been found to spread from person to person, under certain conditions, PRVs may infect humans and cause intense neurological symptoms (9). Recent studies have shown that PRVs have evolved various mechanisms to counteract host immune responses and achieve effective infection (10–17). For example, PRV pUS2 interacts with the ligand-binding domain of STING and recruits TRIM21 to degrade STING (10). PRV pUL13 suppresses the expression of RIG-I and MDA5 by inhibiting the activation of the transcription factor NF-κB, thereby suppressing the host’s antiviral immunity (13). PRV EP0 inhibits the IFN-induced antiviral innate immune response by downregulating the basal expression of IRF9 via transcriptional repression (16). However, it is still unclear whether the PRV proteins target the cGAS-STING axis for immune evasion.

The DNA polymerase processing factor encoded by the *Ul42* gene is one of the components of DNA polymerase in PRV and HSV-1, which is an essential protein for virus replication (18, 19). PRV pUL42 is a continuous synthesis factor of DNA polymerase, which can activate its ability to continuously synthesize viral genomes by binding to the catalytic subunit pUL30 of DNA polymerase (19). Recently, PRV pUL42 was found to induce ubiquitination degradation of p65 by upregulating cytokine signaling inhibitor 1 (SOCS1) to prevent excessive inflammatory response during PRV infection (20). In addition, PRV pUL42 binds to IFN-stimulated response element (ISRE) through four conserved DNA-binding sites, which prevents the binding of IFN-stimulated gene factor 3 (ISGF3) to ISRE and leads to IFN-induced transcriptional disruption (21). However, it is still unknown whether pUL42 regulates the cGAS-STING axis. In this study, we found that PRV pUL42 interacts with the DNA-binding domain of cGAS to inhibit its binding to dsDNA, which subsequently inhibits the dimerization and oligomerization activation of cGAS. We also found that pUL42 interacts with cGAS, which subsequently leads to the suppression of type I IFN production and promotes viral replication. These findings support the notion that pUL42 is a pivotal inhibitor of the cGAS-STING axis, revealing a new mechanism by which PRV-encoded protein pUL42 plays a role in antagonizing host antiviral responses.

## RESULTS

### PRV pUL42 inhibits the cGAS-STING signaling pathway

**Fig 1:**
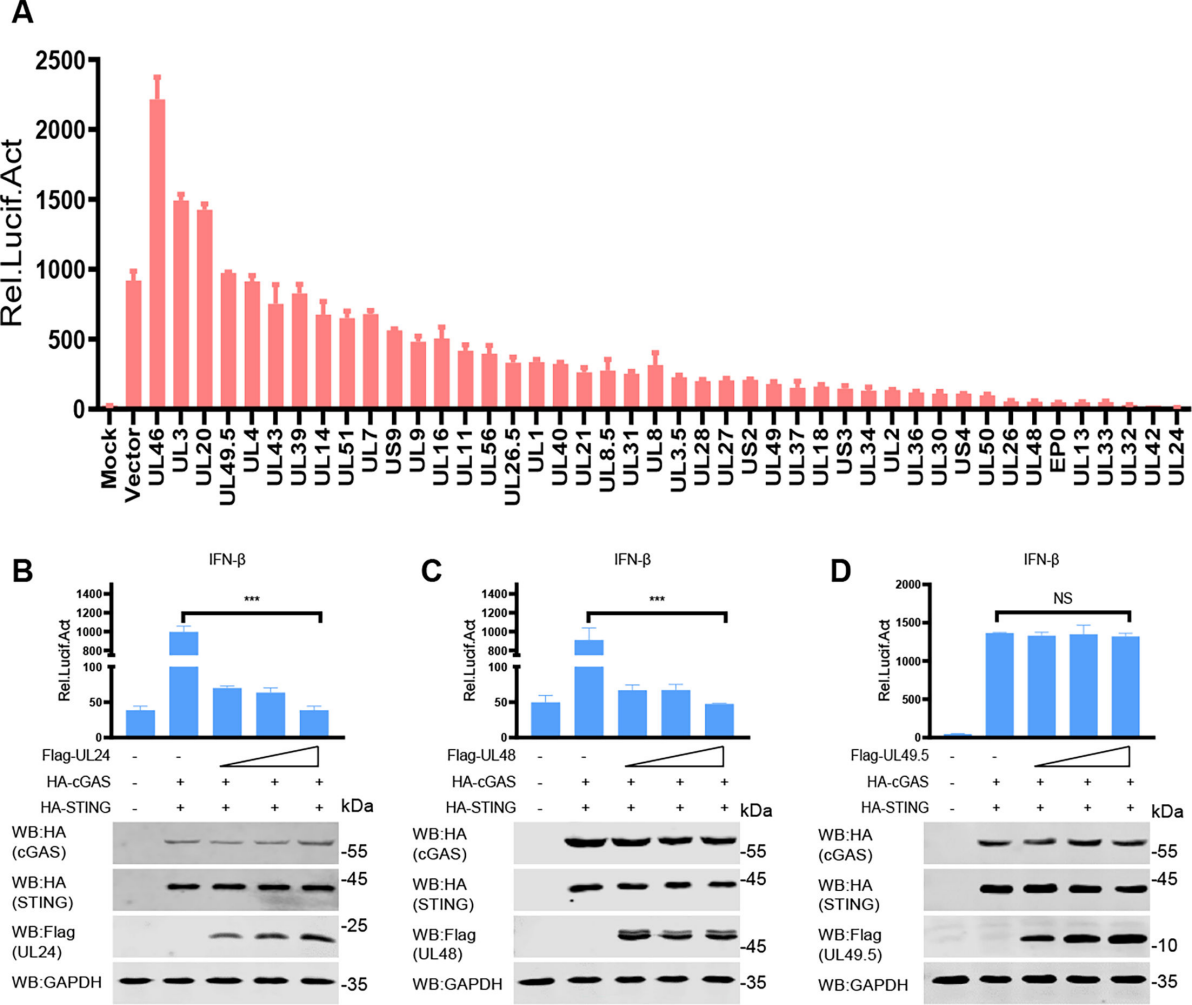
Screening for PRV-encoded proteins that regulate the cGAS-STING signaling pathway. (**A**) HEK293T cells were co-transfected with plasmids expressing cGAS (50 ng), STING (50 ng), the IFN-β reporter (100 ng), pRL-TK reporter (10 ng), and the indicated plasmids encoding PRV proteins (400 ng), respectively. At 24 hpt, the cells were harvested to detect luciferase activity. (**B–D**) HEK293T cells were transfected with an IFN-β luc reporter (100 ng) and a Renilla-TK reporter (10 ng), two plasmids expressing HA-cGAS (50 ng) and HA-STING (50 ng), respectively, together with different amounts (0, 100, 200, and 400 ng) of a plasmid expressing pUL24, pUL48, or pUL49.5 for 24 h. And then the cells were harvested to detect the luciferase activities (upper panels). The HA-tagged cGAS and STING, Flag-tagged pUL24, pUL48, and pUL49.5 proteins, and GAPDH were detected by Western blotting (lower panels). NS, not significant (*P* > 0.05), ***P < 0.001 (two-tailed Student’s t-test).

The cGAS-STING signaling pathway plays an important role in sensing cytoplasmic DNA to induce type I IFN production to trigger host antiviral immune responses (1). Previous research results have shown that PRV infection inhibits cGAS-STING-mediated IFN production (22). To identify which PRV-encoded protein can inhibit the promoter activity of IFN-β induced by cGAS-STING, we conducted an unbiased screening to evaluate the effects of 44 PRV-encoded proteins on the regulation of type I IFN production. We found that pUL24, pUL42, and pUL32 exhibited the most significant inhibitory effects (Fig. 1A). To further validate the effectiveness of the screening system, three PRV proteins (pUL24, pUL48, and pUL49.5) were selected for further evaluation of their inhibitory effects. HEK293T cells were transfected with an IFN-β-luciferase reporter, an internal control Renilla-TK Luc, two plasmids expressing cGAS and STING, together with different doses of a plasmid expressing pUL24, pUL48, or pUL49.5, respectively. As shown in Fig. 1B through D, the ectopic expression of pUL24 and pUL48 inhibited cGAS-STING-induced IFN-β promoter activity in a dose-dependent manner, while pUL49.5 had no effect (Fig. 1B through D). These results demonstrated that our unbiased screening assay is effective.

**Fig 2:**
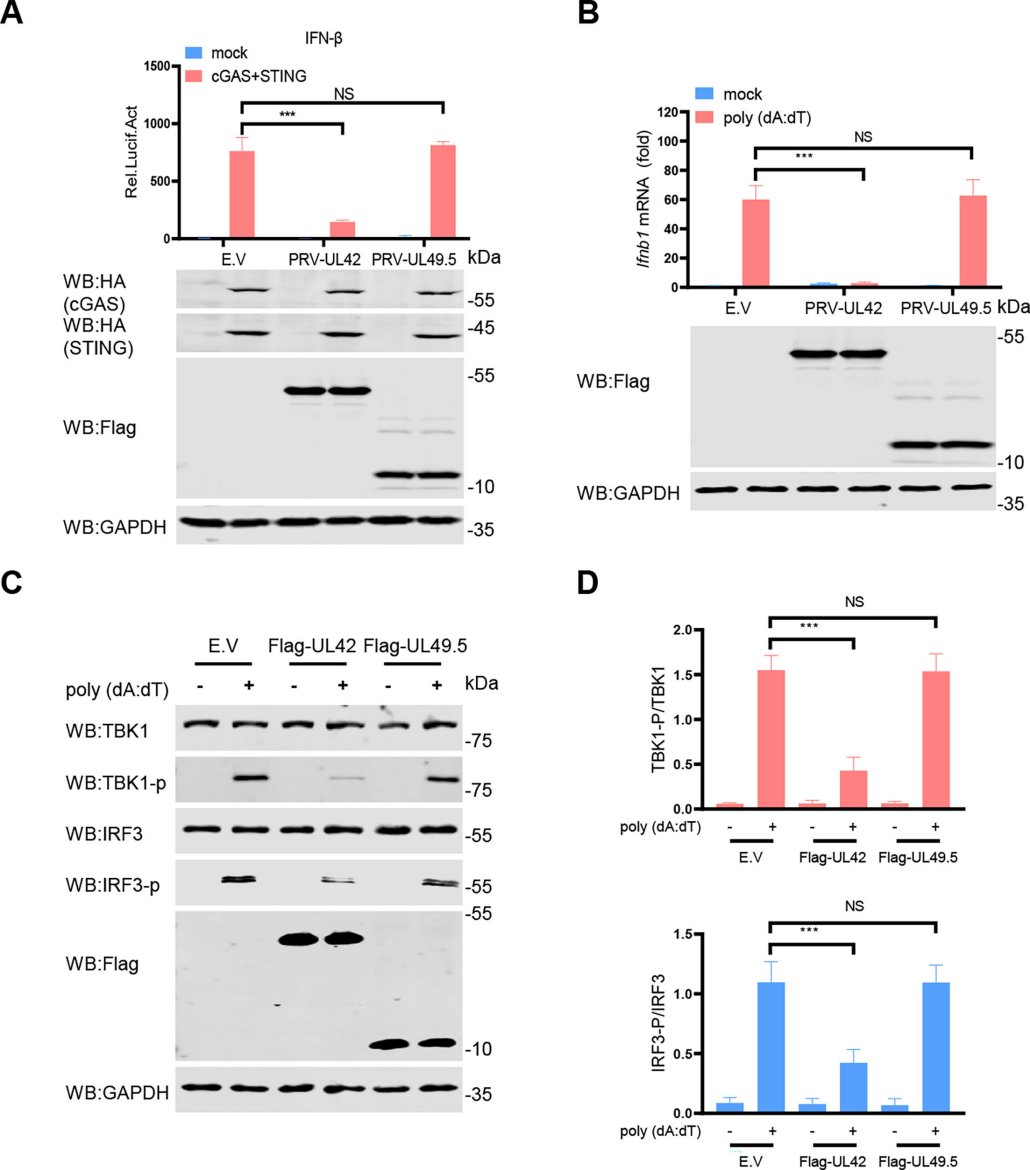
PRV pUL42 suppresses type I IFN production by inhibiting the cGAS-STING signaling pathway. (**A**) Detection of luciferase activity of IFN-β promoter reporter in HEK293T cells induced by cGAS and STING upon pUL42 or pUL49.5 overexpression. HEK293T cells were co-transfected with 400 ng of empty vector or plasmids expressing PRV pUL42 or PRV pUL49.5, together with 100 ng of an IFN-β luciferase reporter and 10 ng of a plasmid expressing Renilla luciferase (pRL-TK), along with 50 ng of plasmids expressing cGAS and STING for 24 h. And then the cells were harvested and analyzed for luciferase activities (upper panels). The HA-tagged cGAS and STING, Flag-tagged pUL42, pUL49.5 proteins, and GAPDH were detected by Western blotting (lower panels). (**B**) Detection of the *Ifnb1* mRNA level in HeLa cells induced by poly(dA:dT) upon pUL42 or pUL49.5 overexpression. HeLa cells were co-transfected with 400 ng empty vector or plasmid PRV pUL42 or PRV pUL49.5. 24 h later, cells were transfected with 1 µg/mL of poly(dA:dT) for 12 h, and then qPCR was performed to analyze *Ifnb1* mRNA level (upper panels). The Flag-tagged pUL42, pUL49.5 proteins, and GAPDH were detected by Western blotting (lower panels). (**C**) Immunoblot analysis of the phosphorylation of TBK1 and IRF3 in HeLa cells induced by poly(dA:dT) upon pUL42 or pUL49.5 overexpression. HeLa cells were transfected with 2 µg of empty vector, a plasmid expressing PRV pUL42 or PRV pUL49.5 for 24 h, and then the cells were transfected with 1 µg/mL of poly(dA:dT) for 12 h. The protein levels of STING, phosphorylated TBK1, TBK1, phosphorylated IRF3, IRF3, Flag-pUL42, pUL49.5, and GAPDH were analyzed by Western blotting. (**D**) The ratio of the intensity value of the TBK1-p/TBK1 and IRF3-p/IRF3 immunoblotting result from (**C**) was quantified by ImageJ. Data represent three independent experiments with three biological replicates or represent three independent experiments with similar results. NS, not significant (*P* > 0.05), ***P < 0.001 (two-tailed Student’s t-test).

**Fig 3:**
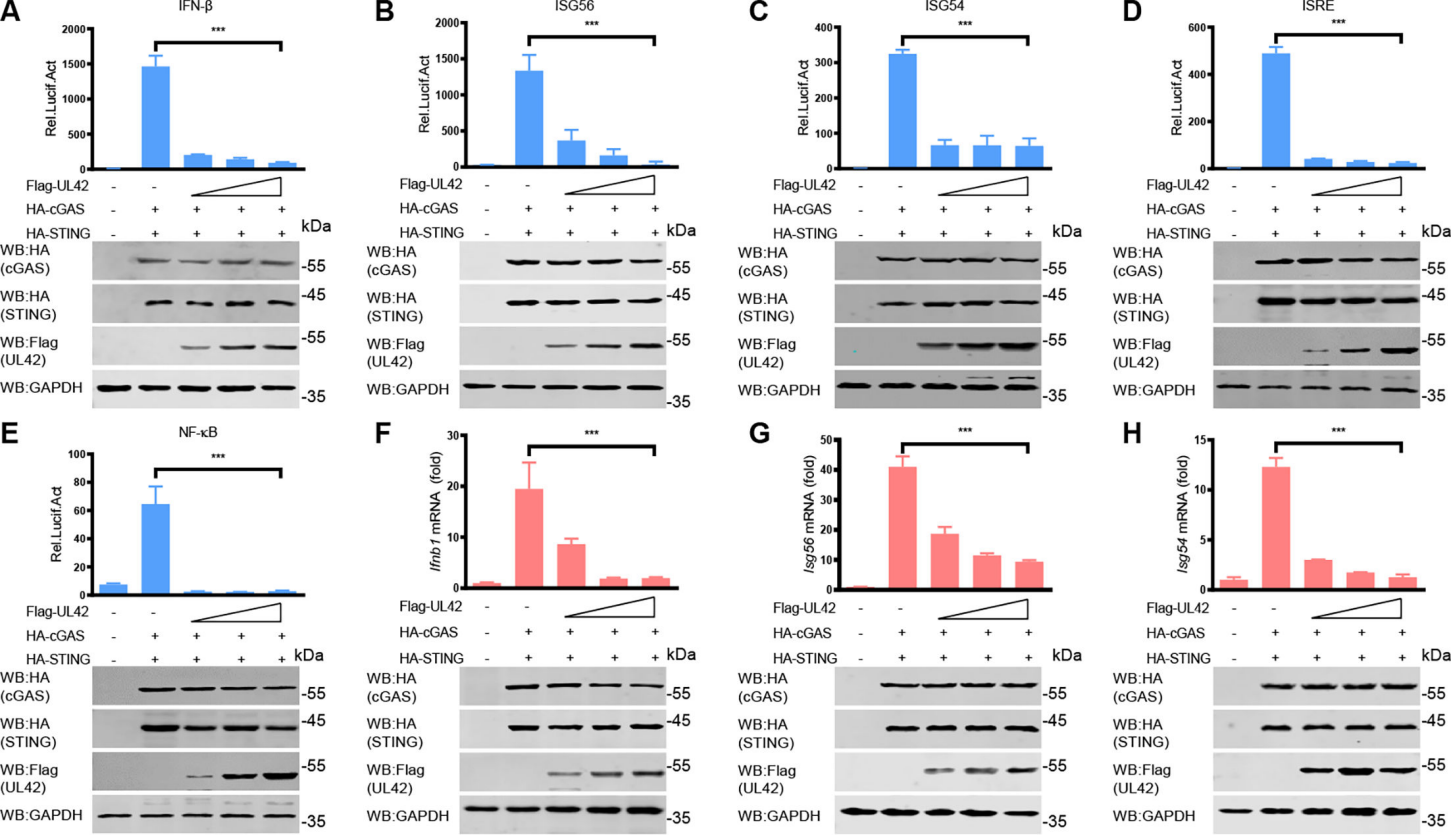
PRV pUL42 suppresses type I IFN production by inhibiting the cGAS-STING signaling pathway. (**A–E**) Luciferase activity of IFN-β (**A**), ISG56 (**B**), ISG54 (**C**), ISRE (**D**), or NF-κB (**E**) promoter reporter in HEK293T cells induced by cGAS and STING upon PRV pUL42 overexpression. HEK293T cells were transfected with an IFN-β (**A**), ISG56 (**B**), ISG54 (**C**), ISRE (**D**), or NF-κB (**E**) luciferase reporter (100 ng/each) and a Renilla-TK reporter (10 ng), and plasmids expressing HA-cGAS (50 ng) and HA-STING (50 ng), together with different amounts (0, 100, 200, and 400 ng) of a plasmid expressing Flag-pUL42 for 24 h, and then luciferase activities were analyzed (upper panels). The HA-tagged cGAS and STING, Flag-tagged pUL42 proteins, and GAPDH were detected by Western blotting (lower panels). (**F–H**) The *Ifnb1*, *Isg56*, and *Isg54* mRNA levels in HEK293T cells induced by cGAS and STING upon PRV pUL42 overexpression. HEK293T cells were transfected with plasmids expressing HA-cGAS (50 ng) and HA-STING (50 ng), together with different amounts (0, 100, 200, and 400 ng) of a plasmid expressing Flag-pUL42 for 24 h. The mRNA levels of *Ifnb1* (**F**), *Isg56* (**G**), and *Isg54* (**H**) in the HEK293T cells were analyzed by qPCR (upper panels). The HA-tagged cGAS, STING, Flag-tagged pUL42, and GAPDH were detected by Western blotting (lower panels). The data represent three independent experiments with three biological replicates or represent three independent experiments with similar results. ***P < 0.001 (two-tailed Student’s t-test).

In our impartial screening system, PRV pUL24 exhibits the most pronounced influence on the activity of the IFN-β promoter induced by cGAS-STING, followed by pUL42. Furthermore, numerous studies have documented the inhibitory role of pUL24 on the cGAS-STING signaling pathway (23–25). A previous study showed that PRV pUL42 can inhibit virus infection-induced type I IFN signaling pathway (21), and HSV-1 pUL42 inhibits the production of type I IFN by inhibiting phosphorylation of IRF3 (26). However, the inhibition effect of PRV pUL42 on the production of type I IFN has not been studied. To test the effect of PRV pUL42 on the production of type I IFN, HEK293T cells were transfected with a plasmid encoding pUL42 or pUL49.5 as a negative control, together with an IFN-β-luciferase reporter, a pRL-TK, along with plasmids expressing cGAS and STING for 24 h. As shown in Fig. 2A, ectopic expression of pUL42 inhibited cGAS-STING-induced IFN-β promoter activity, while pUL49.5 had no effect (Fig. 2A). Additionally, we noticed that pUL42 inhibited the activities of these promoters of IFN-β (Fig. 3A), ISG56 (Fig. 3B), ISG54 (Fig. 3C), ISRE (Fig. 3D), and nuclear factor kappa B (NF-κB; Fig. 3E). To further analyze the inhibitory effect of pUL42 on type I IFN production, HeLa cells were transfected with a plasmid-encoding pUL42 or pUL49.5. At 24 h post-transfection (hpt), the cells were stimulated with poly(dA:dT), a simulation dsDNA sequence to activate the cGAS-STING signaling pathway, for another 12 h, and then the mRNA level of *Ifnb1* was detected by qPCR. As shown in Fig. 2B, ectopically expressed pUL42 significantly decreased the mRNA levels of *Ifnb1* induced by poly(dA:dT), but not pUL49.5. Consistently, we also found that pUL42 inhibited the mRNA levels of *Ifnb1* (Fig. 3F), *Isg56* (Fig. 3G), and *Isg54* (Fig. 3H) induced by cGAS-STING. These data suggest PRV pUL42 is a potent inhibitor of type I IFN production.

Phosphorylation of TBK1 and IRF3 is an important marker of cGAS-STING signaling pathway activation. To test whether pUL42 affects the phosphorylation of TBK1 and IRF3, HeLa cells were transfected with a plasmid encoding pUL42 or pUL49.5 for 24 h, and then transfected with poly(dA:dT) for another 12 h. The phosphorylation levels of IRF3 (Ser396) and TBK1 (Ser172) were detected. As shown in Fig. 2C and D, pUL42 significantly inhibited the phosphorylation of IRF3 and TBK1 induced by poly(dA:dT), while pUL49.5 had no effect.

### Knockdown of the expression of *Ul42* promotes type I IFN production induced by PRV

**Fig 4:**
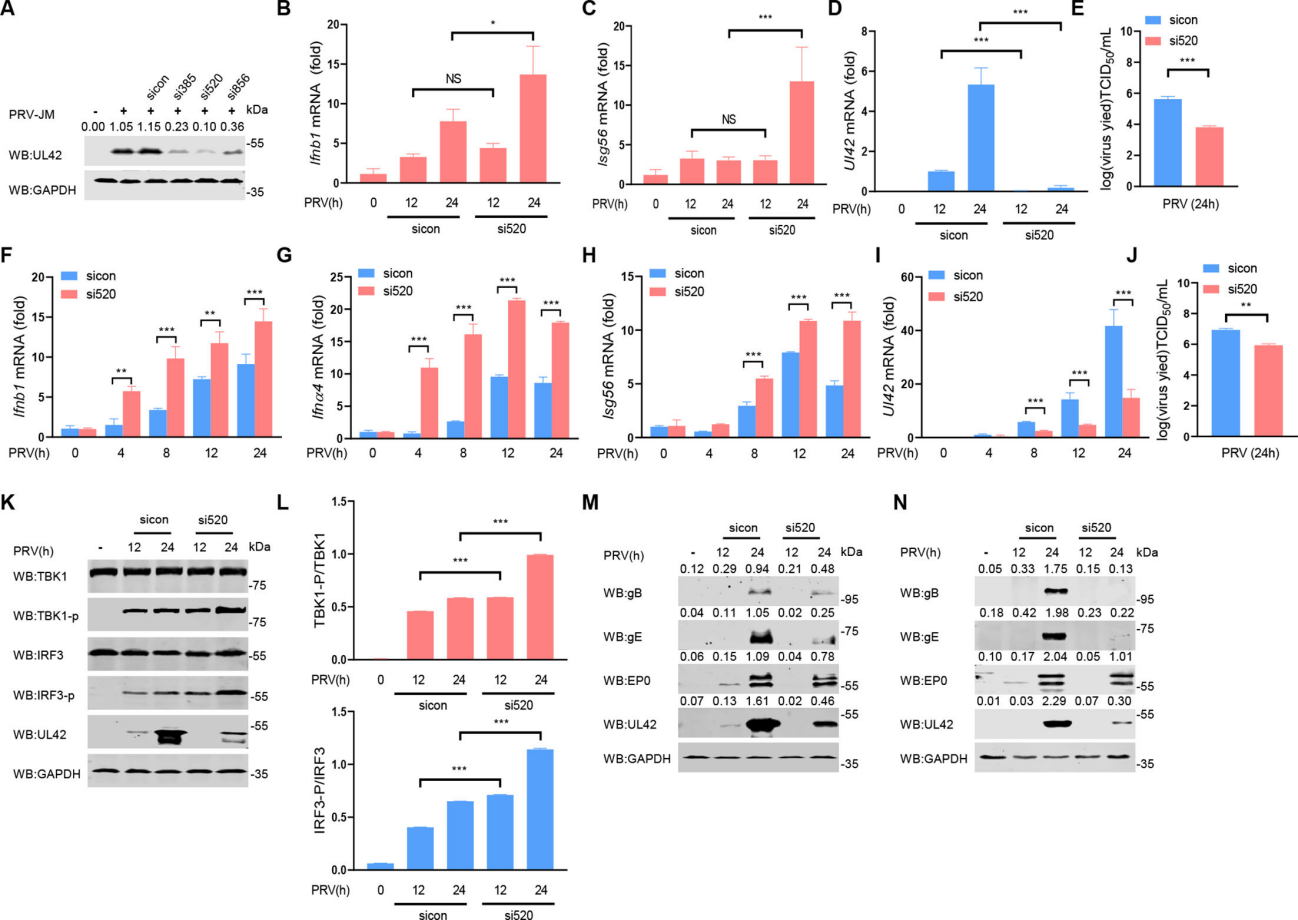
Knockdown of *Ul42* expression of PRV induces higher type I IFN production. (**A**) The efficiency of siRNAs targeting the *Ul42* gene. PAMs were transfected with siRNA (100 nM/each) targeting the *Ul42* gene, at 48 hpt, and the UL42 and GAPDH were detected by Western blotting. The ratio of the intensity value of the pUL42/GAPDH immunoblotting result was quantified by ImageJ. (**B–E**) Analysis of *Ifnb1* and *Isg56* mRNA expression in PAMs infected with PRV-WT or *Ul42*-knockdown PRV. PAMs were transfected with si520 or sicon (100 nM/each) for 48 h, then PAMs were infected with PRV (1 MOI) for 0, 12, or 24 h. The mRNA levels of *Ifnb1* (**B**), *Isg56* (**C**), and *Ul42* (**D**) in cells were detected by qPCR, and the titers of PRV-WT or *Ul42*-knockdown PRV infectious progeny virions were detected (**E**). (**F–J**) Analysis of *Ifnb1*, *Ifnα4,* and *Isg56* mRNA expression in HeLa cells infected with PRV-WT or *Ul42*-knockdown PRV. HeLa cells were transfected with si520 or sicon (100 nM/each) for 24 h, and then HeLa cells were infected with PRV (1 MOI) for 0, 4, 8, 12, or 24 h. The mRNA levels of *Ifnb1* (**F**), *Ifnα4* (**G**), *Isg56* (**H**), and *Ul42* (**I**) in cells were detected by qPCR, and the titers of PRV-WT or *Ul42*-knockdown PRV infectious progeny virions were detected (**J**). (**K**) Immunoblot analysis of the phosphorylation of TBK1 and IRF3 in HeLa cells induced by PRV-WT or *Ul42*-knockdown PRV. HeLa cells were transfected with si520 or sicon (100 nM/each) for 24 h, and then HeLa cells were infected with PRV (1 MOI) for 0, 12, or 24 h. The protein levels of TBK1, phosphorylated TBK1, IRF3, phosphorylated IRF3, pUL42, and GAPDH were analyzed by Western blotting. (**L**) The ratio of the intensity value of the TBK1-p/TBK1 and IRF3-p/IRF3 immunoblotting result from (**K**) was quantified by ImageJ. (**M and N**) Immunoblotting analysis of the effect of *Ul42* knockdown on PRV protein expression. PAMs (**M**) and HeLa cells (**N**) were transfected with si520 or sicon (100 nM/each) for 24 h, and then PAMs (**M**) or HeLa cells (**N**) were infected with PRV (1 MOI) for 0, 12, or 24 h. The protein levels of gB, gE, EP0, pUL42, and GAPDH were analyzed by Western blotting. The ratio of the intensity value of the gB/GAPDH, gE/GAPDH, EP0/GAPDH, and pUL42/GAPDH immunoblotting result was quantified by ImageJ. Data are representative of three independent experiments with three biological replicates (mean ± s.d.). NS, not significant (*P* > 0.05), *P < 0.05, **P < 0.01, ***P < 0.001 (two-tailed Student’s t-test).

To investigate whether *Ul42* affects the production of type I IFN during PRV infection, we used CRISPR-Cas9 and homologous recombination technology to construct an *Ul42*-deficient PRV, but due to the important role of *Ul42* in viral replication, we were unable to obtain *Ul42*-deficient PRV. Then, porcine alveolar macrophages (PAMs) were transfected with small interfering RNA (siRNA) control (sicon) and three siRNAs targeting *Ul42* (si385, si520, and si856) for 48 h and then infected with PRV-JM (multiplicity of infection [MOI] of 1) for 24 h (Fig. 4A). The results showed that the knockdown efficiency of si520 is the highest for pUL42. PAMs were transfected with sicon and si520 for 48 h and then infected with PRV-JM (1 MOI) for 12 h or 24 h. As shown in Fig. 4B and C, we found that knockdown of the expression of *Ul42* resulted in increased mRNA levels of *Ifnb1* and *Isg56* during PRV infection. Consistent with these results, si520 markedly inhibited the mRNA level of *Ul42* during PRV infection (Fig. 4D). The si520 resulted in a decrease of approximately two logs in the viral titers of PRV in PAMs (Fig. 4E).

Previous studies have shown that PRV spreads among populations of different species (27). We further investigated the function of pUL42 in HeLa cells. HeLa cells were transfected with sicon and si520 for 24 h and then infected with PRV-JM (1 MOI) for 0, 4, 8, 12, or 24 h. As shown in Fig. 4F through H, we found that knockdown of *Ul42* resulted in increased mRNA levels of *Ifnb1*, *Ifnα4,* and *Isg56* during PRV infection. Consistent with the results on PAMs, knocking down *Ul42* resulted in a significant decrease of virus titers (Fig. 4I and J). Meanwhile, we found that knockdown of *Ul42* expression significantly facilitated PRV-induced phosphorylation of TBK1 and IRF3 in HeLa cells (Fig. 4K and L). Further research found that knockdown *Ul42* inhibited the protein expression of gB, gE, and EP0 in the process of PRV infection in PAMs (Fig. 4M) and HeLa cells (Fig. 4N). These findings are consistent with previous results that highlight the critical role of *Ul42* in viral replication (19, 28). In summary, we have demonstrated that pUL42 is necessary for virus replication, which also inhibits the production of type I IFN by targeting the cGAS-STING signaling pathway.

### PRV pUL42 interacts with cGAS

**Fig 5:**
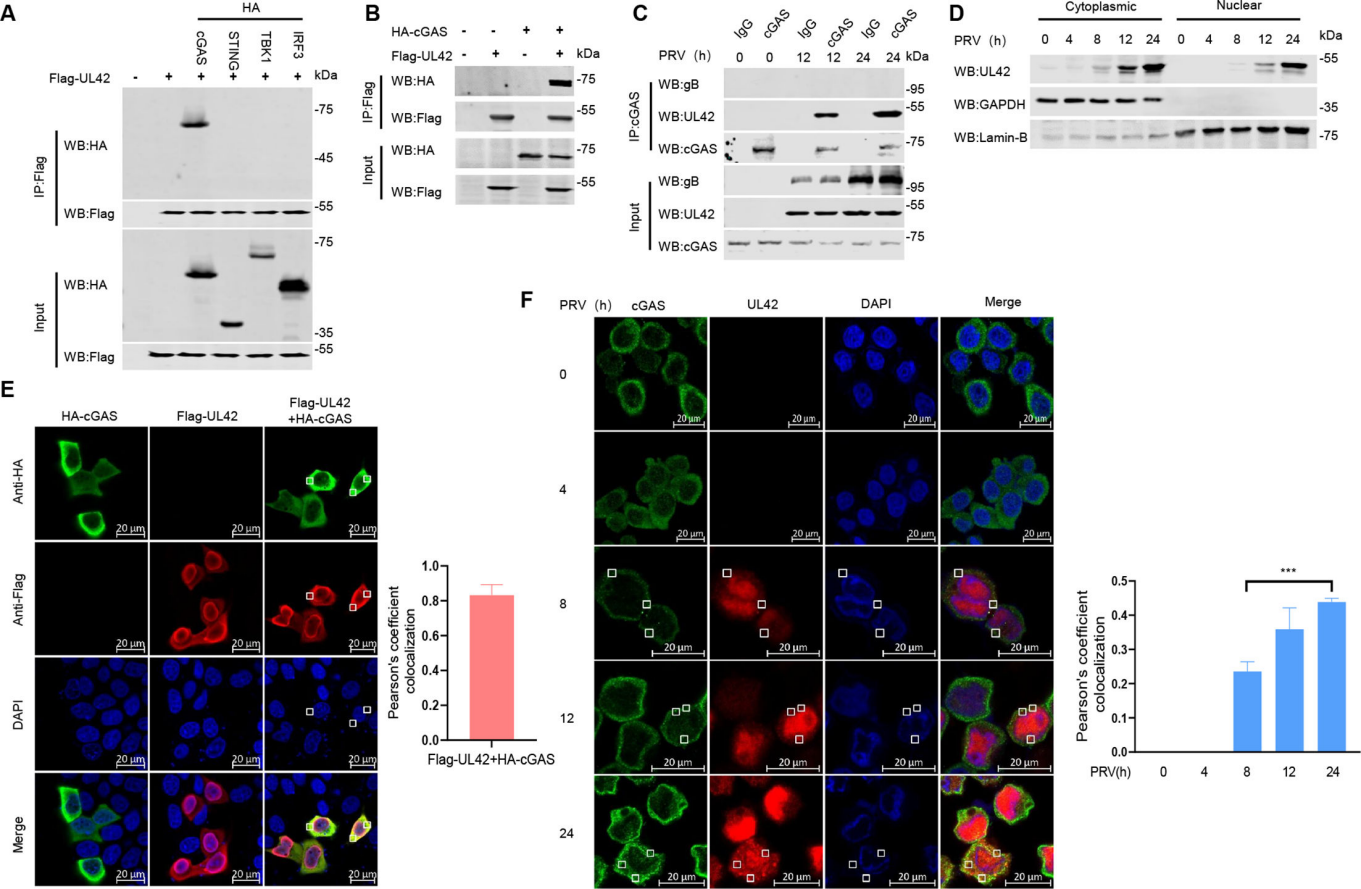
PRV pUL42 interacts with cGAS. (**A**) Co-immunoprecipitation (Co-IP) analysis of the interaction between overexpressed Flag-pUL42 and HA-cGAS, HA-STING, HA-TBK1, or HA-IRF3 in HEK293T cells. HEK293T cells were transfected with plasmids expressing Flag-pUL42 (2 µg) alone or together with a plasmid expressing HA-cGAS, STING, TBK1, and IRF3 (2 µg/each) for 24 h. The cell lysates were harvested and used for the Co-IP. Whole-cell lysates (WCLs) and IP complexes were analyzed by Western blotting using antibodies against Flag or HA. (**B**) Co-IP analysis of the interaction between overexpressed Flag-pUL42 and HA-cGAS in HEK293T cells. HEK293T cells were transfected with 2 µg of plasmids expressing Flag-pUL42, HA-cGAS alone, or both. At 24 hpt, the cell lysates were harvested and used for Co-IP. Co-IP complexes and WCL were analyzed by Western blotting using antibodies against Flag or HA. (**C**) Co-IP analysis of the interaction between pUL42 and cGAS in PAMs infected with PRV. PAMs were infected with PRV (1 MOI) for 0, 12, and 24 h, then Co-IP was performed with anti-cGAS antibody to analyze the interaction of endogenous cGAS and PRV-encoded pUL42. Immunoglobulin G (IgG) was used as a negative control. (**D**) The nuclear translocation of pUL42 in HeLa cells was induced by PRV. HeLa cells were infected with PRV (1 MOI) for 0, 4, 8, 12, or 24 h, and pUL42 in the nuclear and cytoplasmic compartments was detected by Western blotting. Lamin B and GAPDH were used as nuclear and cytoplasmic markers. (**E**) The subcellular localization of overexpressed pUL42 and cGAS in HeLa cells. HeLa cells were transfected with 0.5 µg of plasmids expressing HA-cGAS or Flag-pUL42 alone, or both. At 24 hpt, the subcellular localization of cGAS and pUL42 in the HeLa cells was detected by confocal microscopy. Scale bars, 20 µm. Pearson’s correlation coefficient of the images was analyzed using the Zeiss processing system. (**F**) The subcellular colocalization of pUL42 and endogenous cGAS upon PRV infection in HeLa cells. HeLa cells were infected with PRV (1 MOI) for 0, 4, 8, 12, or 24 h, and the subcellular localization of pUL42 and cGAS in the HeLa cells was detected by confocal microscopy. Scale bars, 20 µm. Pearson’s correlation coefficient of the images was analyzed using the Zeiss processing system. ***P < 0.001 (two-tailed Student’s t-test).

To elucidate the potential molecular mechanism of pUL42 negatively regulating type I IFN production, immunoprecipitation assays were performed. As shown in Fig. 5A and B, we found that pUL42 interacted with cGAS in HEK293T cells, but not STING, TBK1, or IRF3. In addition, we found that endogenous cGAS interacted with pUL42 in PRV-infected PAMs at 12 h and 24 h, but not gB (Fig. 5C). Previous studies have shown that pUL42 relies on nuclear location signals (NLS) to transport to the nucleus and participates in DNA replication along with other viral proteins (29), and cGAS mainly recognizes viral-dsDNA in the cytoplasm. Western blotting results clearly demonstrated that pUL42 exists in the cytoplasm within 8–24 h of PRV infection (Fig. 5D). We also found pUL42 colocalized with cGAS in the cytoplasm when the two proteins were co-expressed in HeLa cells, with Pearson coefficients of about 0.8 (Fig. 5E). In addition, we noticed that endogenous cGAS colocalized with pUL42 in the cytoplasm in PRV-infected HeLa cells, with Pearson coefficients of about 0.4 at 24 h (Fig. 5F). These results indicate that pUL42 targets cGAS to inhibit type І IFN production in cytoplasm.

**Fig 6:**
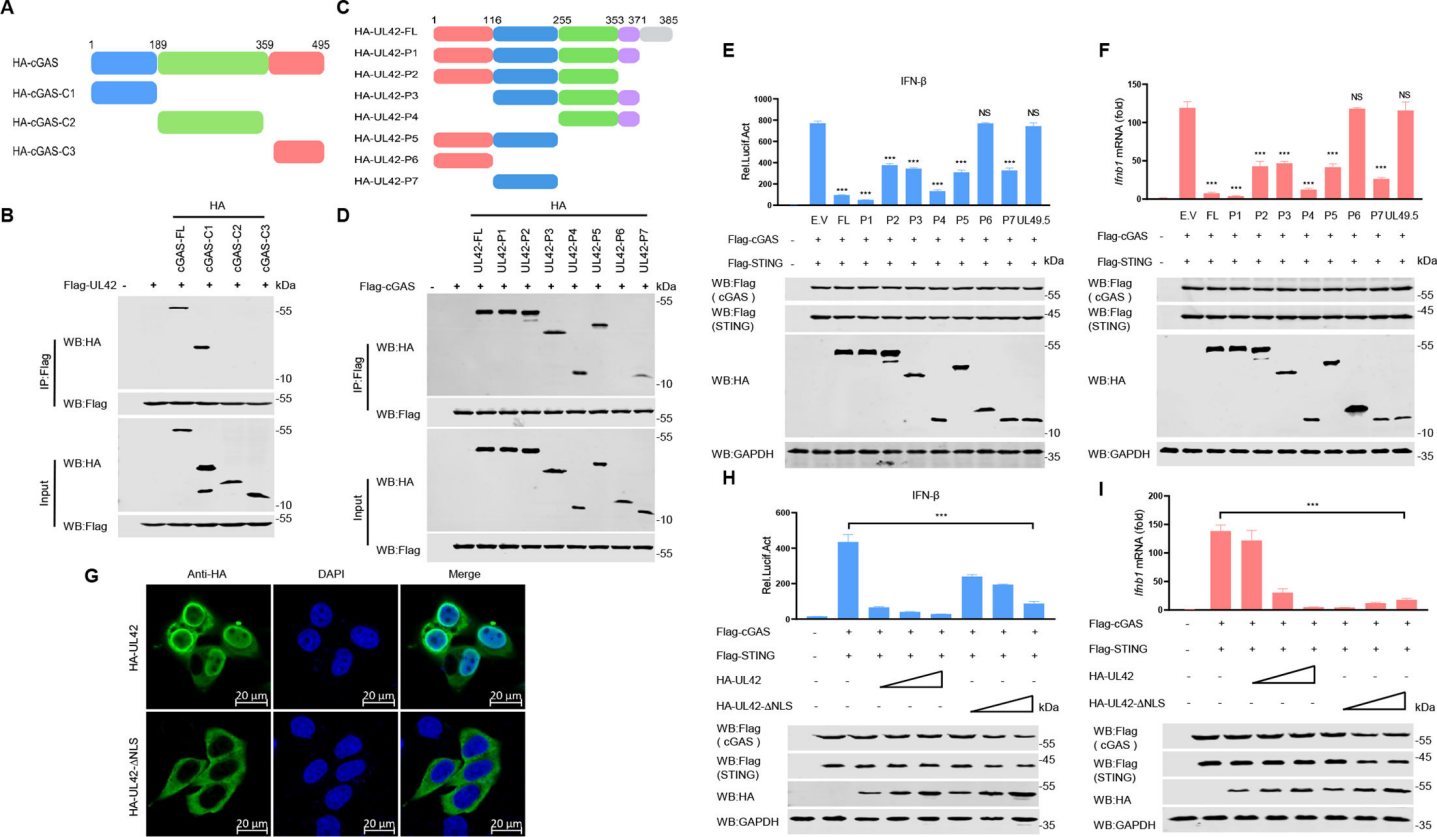
The 116–353 aa of pUL42 interacts with DNA-binding domain of cGAS. (**A**) Schematic diagram illustrating the truncated mutants of cGAS. (**B**) Co-IP analysis of the interaction between overexpressed pUL42 and cGAS or its truncated mutants in HEK293T cells. HEK293T cells were transfected with 2 µg of a plasmid expressing Flag-pUL42 alone or together with plasmids expressing HA-cGAS and its truncated mutants. At 24 hpt, the cell lysates were harvested and used for Co-IP. Co-IP and input complexes were analyzed by Western blotting using antibodies against Flag or HA. (**C**) Schematic diagram illustrating the truncated mutants of pUL42. (**D**) Co-IP analysis of the interaction between overexpressed cGAS and pUL42 or its truncated mutants in HEK293T cells. HEK293T cells were transfected with 2 µg of a plasmid expressing Flag-cGAS alone or together with plasmids expressing HA-pUL42 and its truncated mutants (2 µg/each). At 24 hpt, the cell lysates were harvested and used for Co-IP. Co-IP and input complexes were analyzed by Western blotting using antibodies against Flag or HA. (**E**) Luciferase activity of IFN-ꞵ promoter reporter in HEK293T cells induced by cGAS and STING upon overexpression of pUL42, its truncation mutants, or pUL49.5. HEK293T cells were transfected with an IFN-ꞵ luciferase reporter (100 ng), a Renilla-TK reporter (10 ng), and plasmids expressing pUL42 and its truncation mutants (400 ng/each) along with plasmids expressing cGAS (50 ng) and STING (50 ng) for 24 h. The cells were collected to detect luciferase activity (upper panels). GAPDH, Flag-tagged cGAS and STING, and HA-tagged pUL42 and its truncation mutants were verified by Western blotting (lower panels). (**F**) *Ifnb1* mRNA levels in HEK293T cells induced by cGAS and STING upon overexpression of pUL42 or its truncation mutants. HEK293T cells were transfected with plasmids expressing pUL42 or its truncation mutants (400 ng/each) along with plasmids expressing cGAS (50 ng) and STING (50 ng) for 24 h. The mRNA levels of *Ifnb1* in the HEK293T cells were analyzed by qPCR (upper panels). GAPDH, Flag-tagged cGAS and STING, and HA-tagged pUL42 and its truncation mutants were verified by Western blotting (lower panels). (**G**) The subcellular localization of overexpressed pUL42 or pUL42 lacking NLS (pUL42-∆NLS). HeLa cells were transfected with 1 µg of a plasmid expressing pUL42 or pUL42-∆NLS. At 24 hpt, the subcellular localization of pUL42 or pUL42-∆NLS in the HeLa cells was detected by confocal microscopy. Scale bars, 20 µm. (**H**) The effect of the pUL42 or pUL42-∆NLS on the luciferase activity of IFN-β promoter reporter in HEK293T cells induced by cGAS and STING. HEK293T cells were transfected with an IFN-β luciferase reporter (100 ng) and a Renilla-TK reporter (10 ng), and plasmids expressing Flag-cGAS (50 ng) and Flag-STING (50 ng), together with different amounts (0, 100, 200, and 400 ng) of a plasmid expressing HA-UL42 or HA-UL42-∆NLS for 24 h, and then luciferase activities were analyzed (upper panels). The Flag-tagged cGAS and STING, HA-tagged UL42 or HA-UL42-∆NLS proteins, and GAPDH were detected by Western blotting (lower panels). (**I**) The effect of the pUL42 or pUL42-∆NLS on the *Ifnb1* mRNA level in HEK293T cells induced by cGAS and STING. HEK293T cells were transfected with plasmids expressing Flag-cGAS (50 ng) and Flag-STING (50 ng), together with different amounts (0, 100, 200, and 400 ng) of a plasmid expressing HA-UL42 or HA-UL42-∆NLS for 24 h, and then qPCR was performed to analyze *Ifnb1* mRNA level (upper panels). The Flag-tagged cGAS and STING, HA-tagged UL42 or HA-UL42-∆NLS proteins, and GAPDH were detected by Western blotting (lower panels). NS, not significant (*P* > 0.05), ***P < 0.001 (two-tailed Student’s t-test).

The cGAS protein consists of an unstructured N-terminus and a highly conserved C-terminus (30). The N-terminus of cGAS has been predicted to bind to DNA (31). The C-terminal domain of cGAS contains two highly conserved motifs, including a nucleotide transferase (NTase) core domain and a Mab21 domain with zinc-ribbon insertion (32, 33). To investigate which domain is required for the interaction between pUL42 and cGAS, three plasmids expressing three truncated mutants of cGAS (cGAS-C1, cGAS-C2, and cGAS-C3) were constructed (Fig. 6A). Co-immunoprecipitation (Co-IP) results showed that the DNA-binding domain at the N-terminus of cGAS was required for its interaction with pUL42 (Fig. 6B). To elucidate the functional domain by which pUL42 negatively regulates type I IFN production, seven truncated mutants of pUL42, including pUL42-P1 (aa 1–371), pUL42-P2 (aa 1–353), pUL42-P3 (aa 116–371), pUL42-P4 (aa 255–371), pUL42-P5 (aa 1–255), pUL42-P6 (aa 1–116), and pUL42-P7 (aa 116–255) were constructed (Fig. 6C). We confirmed that the 116–353 aa of pUL42 interacted with cGAS (Fig. 6D). As shown in Fig. 6E and F, ectopically expressed pUL42, pUL42-P1, pUL42-P2, pUL42-P3, pUL42-P4, pUL42-P5, and pUL42-P7 significantly inhibited the IFN-β-promoter activation and mRNA level of *Ifnb1* induced by cGAS and STING, but not pUL42-P6 and pUL49.5. We also found that the 116–255 aa and the 255–371 aa of pUL42 independently interact with cGAS. These interactions result in truncated mutants of pUL42 exhibiting differential impairments in IFN-β-promoter activation and mRNA level of *Ifnb1* induced by cGAS and STING. Notably, pUL42-P4 demonstrates an inhibitory effect comparable to that of pUL42-FL and pUL42-P1, whereas pUL42-P5 and pUL42-P7 do not exhibit such inhibitory activity (Fig. 6D through F). These results suggested that 116–353 aa of pUL42 is required for its inhibition of type I IFN production. Taken together, we demonstrated that the 116–353 aa of pUL42 interacts with the N-terminal of cGAS, which is dependent on the 116–353 aa of pUL42 to inhibit type I IFN production.

Previous studies have identified that pUL42 contains a functional and transferable bipartite NLS at amino acids 354–370 (34). To investigate whether pUL42 exerts its primary inhibitory function in the cytoplasm, a plasmid expressing a truncated mutant of pUL42 lacking NLS (aa 353–371) (pUL42-∆NLS) was constructed. We found that wild-type pUL42 was primarily located in both the nucleus and cytoplasm, while pUL42-∆NLS was completely localized in the cytoplasm of HeLa cells (Fig. 6G). As shown in Fig. 6H and I, ectopically expressed pUL42 and pUL42-∆NLS significantly inhibited the activation of the IFN-β-promoter and the mRNA level of *Ifnb1* induced by cGAS-STING, we noticed that pUL42-∆NLS has the capacity to mitigate some of the effects of pUL42 on the promoter activity of IFN-β induced by the cGAS and STING. This suggests that pUL42 may interact with additional nuclear proteins to exert an inhibitory influence on type І IFN production. Furthermore, these findings corroborate the notion that pUL42 is involved in the suppression of type І IFN production within the cytoplasmic compartment.

### The DNA-binding sites of PRV pUL42 are required for inhibiting type I IFN production

**Fig 7:**
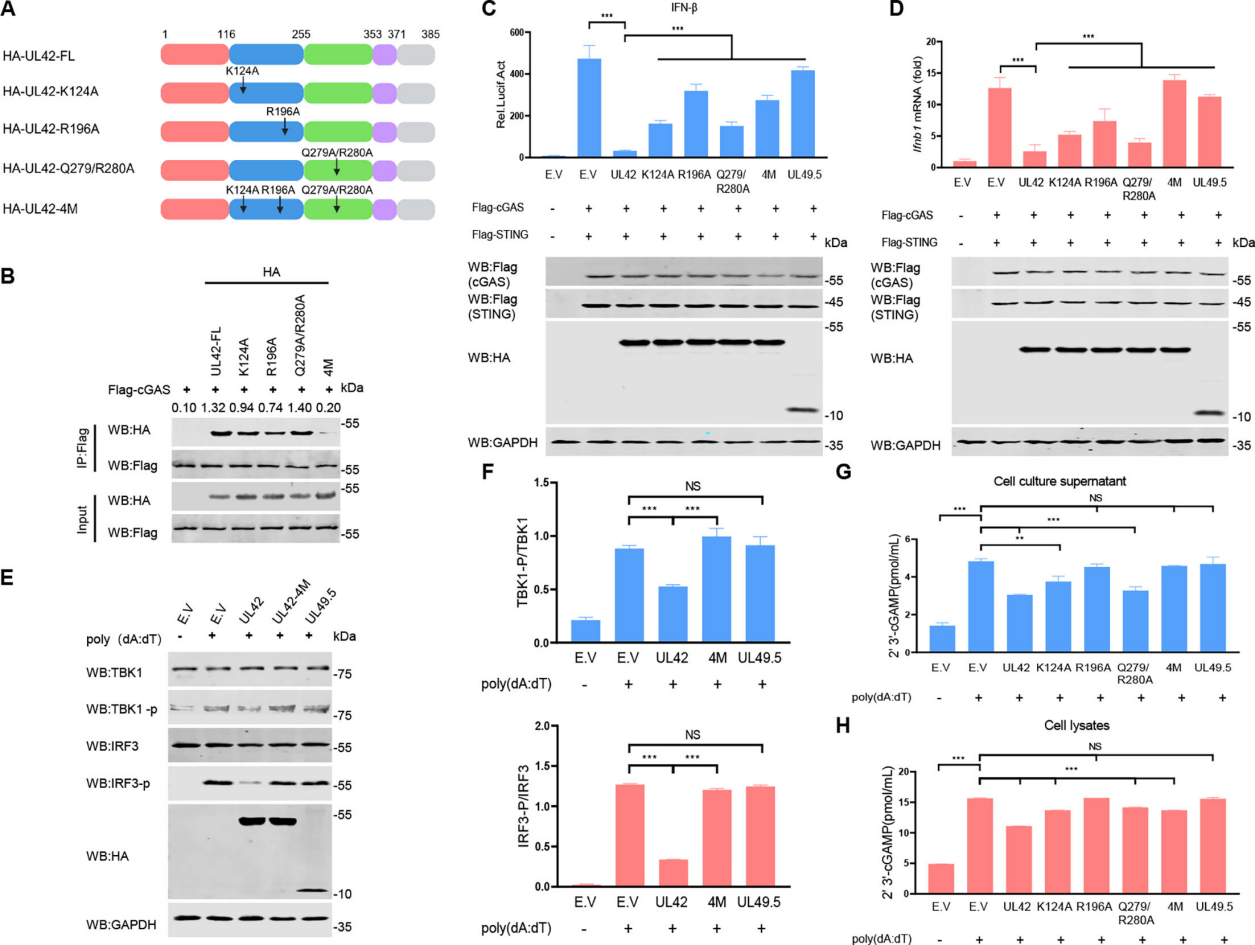
The four amino acid residues in PRV pUL42 are necessary for its inhibitory function on type I IFN production. (**A**) Schematic diagram illustrating the truncated mutants and the DNA-binding site mutants of PRV-pUL42. The four DNA-binding sites of PRV-UL42 are marked by arrows. (**B**) The effect of the DNA-binding sites of PRV pUL42 on the interaction of cGAS and pUL42. HEK293T cells were transfected with a plasmid expressing Flag-cGAS (2 µg) alone or together with a plasmid expressing HA-pUL42, pUL42-K124A, pUL42-R196A, pUL42-Q279A/R280A, or pUL42-4M (2 µg/each) for 24 h. The cell lysates were harvested and used for the Co-IP. Whole-cell lysate (WCL) and IP complexes were analyzed by western blotting using antibodies against Flag or HA. The ratio of the intensity value of the HA-UL42/Flag-cGAS immunoblotting result was quantified by ImageJ. (**C**) The effect of the DNA-binding sites of PRV pUL42 on the luciferase activity of IFN-β promoter reporter in HEK293T cells induced by cGAS and STING. HEK293T cells were co-transfected with 400 ng of empty vector or a plasmid expressing pUL42, pUL42-K124A, pUL42-R196A, pUL42-Q279A/R280A, pUL42-4M, or pUL49.5, together with 100 ng of an IFN-β luciferase reporter and 10 ng of a plasmid expressing pRL-TK, along with 50 ng of plasmids expressing cGAS and STING for 24 h. And then the cells were harvested and analyzed for luciferase activities (upper panels). The Flag-tagged cGAS and STING, HA-tagged pUL42, pUL42-K124A, pUL42-R196A, pUL42-Q279A/R280A, pUL42-4M, or pUL49.5 proteins and GAPDH were detected by Western blotting (lower panels). (**D**) The effect of the DNA-binding sites of PRV pUL42 on the *Ifnb1* mRNA levels in HEK293T cells induced by cGAS and STING. HEK293T cells were co-transfected with 400 ng of empty vector or a plasmid expressing pUL42, pUL42-K124A, pUL42-R196A, pUL42-Q279A/R280A, pUL42-4M, or pUL49.5, along with 50 ng of plasmids expressing cGAS and STING for 24 h, and then qPCR was performed to analyze *Ifnb1* mRNA levels (upper panels). The Flag-tagged cGAS and STING, HA-tagged pUL42, pUL42-K124A, pUL42-R196A, pUL42-Q279A/R280A, pUL42-4M, and pUL49.5 proteins and GAPDH were detected by Western blotting (lower panels). (**E**) The effect of the DNA-binding sites of PRV pUL42 for pUL42-mediated inhibition of phosphorylation of TBK1 and IRF3 in HeLa cells induced by poly(dA:dT). HeLa cells were transfected with 2 µg of empty vector, a plasmid expressing PRV pUL42, pUL42-4M, or PRV pUL49.5 for 24 h, and then the cells were transfected with 1 µg/mL of poly(dA:dT) for 12 h. The protein levels of phosphorylated TBK1, TBK1, phosphorylated IRF3, IRF3, HA-pUL42, pUL42-4M, pUL49.5, and GAPDH were analyzed by Western blotting. (**F**) The ratio of the intensity value of the TBK1-p/TBK1 and IRF3-p/IRF3 immunoblotting result was quantified by ImageJ. (**G and H**) HeLa cells were transfected with 2 µg of empty vector, a plasmid expressing PRV pUL42, pUL42-K124A, pUL42-R196A, pUL42-Q279/R280A, pUL42-4M, or PRV pUL49.5 for 24 h, and then the cells were transfected with 1 µg/mL of poly(dA:dT) for 12 h. The cell supernatant and cell lysate were collected, and the content of cGAMP was detected. The data represent three independent experiments with three biological replicates or represent three independent experiments with similar results. NS, not significant (*P* > 0.05), **P < 0.01, ***P < 0.001 (one-way ANOVA).

Previous research results have shown that the four conserved arginine residues of HSV-1 pUL42 are crucial for interacting with DNA, whereas pUL42 binds to DNA through several positively charged amino acids on its surface (21, 35). We performed sequence alignment and found that the Lys124, Arg196, Gln279, and Arg280 in PRV pUL42, corresponding to the four arginine residues of HSV-1 pUL42 (Fig. 7A). To examine whether the DNA-binding sites of pUL42 are necessary for interaction with cGAS, four mutants of pUL42 DNA-binding sites (K124A, R196A, Q279A/R280A, and 4M) were constructed (Fig. 7A). Co-IP results showed that pUL42-4M (lacking all four DNA-binding sites K124A/R196A/Q279A/R280A) completely lost the ability to interact with cGAS, while other mutants lacking one to three DNA-binding sites retained or partially retained the ability to interact with cGAS (Fig. 7B). Consistently, pUL42-4M completely lost the ability to inhibit the activation of IFN-β-promoter and increasing mRNA levels of *Ifnb1* induced by cGAS-STING, while other mutants of pUL42 retained or partially retained the ability to inhibit the activation of IFN-β-promoter and increasing mRNA levels of *Ifnb1* induced by cGAS-STING (Fig. 7C and D). pUL49.5 served as a negative control. To test whether four DNA-binding sites of pUL42 affect the ability to inhibit the phosphorylation of TBK1 and IRF3 induced by poly(dA:dT), HeLa cells were transfected with a plasmid encoding pUL42, pUL42-4M, or pUL49.5 for 24 h, and the cells were then transfected with poly(dA:dT) for another 12 h. The phosphorylation levels of IRF3 (Ser396) and TBK1 (Ser172) were detected. As shown in Fig. 7E–F, pUL42-4M completely lost the ability to inhibit the phosphorylation of IRF3 and TBK1 induced by poly(dA:dT), pUL49.5 as a negative control.

As a second messenger, cGAMP can be catalyzed by cGAS in the cytoplasm to directly bind and activate the STING-mediated type I IFN production, playing an important role in host antiviral immune responses. To investigate whether pUL42 has the effect on the cGAMP production induced by poly(dA:dT), HeLa cells were transfected with a plasmid encoding pUL42, pUL42-K124A, pUL42-R196A, pUL42-Q279A/R280A, pUL42-4M, or pUL49.5 for 24 h, and the cells were then transfected with poly(dA:dT) for another 12 h. The cell supernatant and cell lysates were collected, and the content of cGAMP was detected. As shown in Fig. 7G and H, pUL42 significantly inhibited the cGAMP production induced by poly(dA:dT), whereas pUL42-R196A and pUL42-4M completely lost the ability to inhibit the cGAMP production induced by poly(dA:dT). As a negative control, pUL49.5 did not inhibit the cGAMP production induced by poly(dA:dT). These results have demonstrated that pUL42 significantly inhibited the cGAMP production induced by poly(dA:dT) and associated with its DNA-binding sites. Taken together, we have demonstrated that pUL42 interacts with cGAS through its DNA-binding sites, which is important for pUL42 function to inhibit the cGAS-STING-mediated type I IFN production.

### PRV pUL42 binds to cGAS and prevents the association of cGAS with DNA

**Fig 8:**
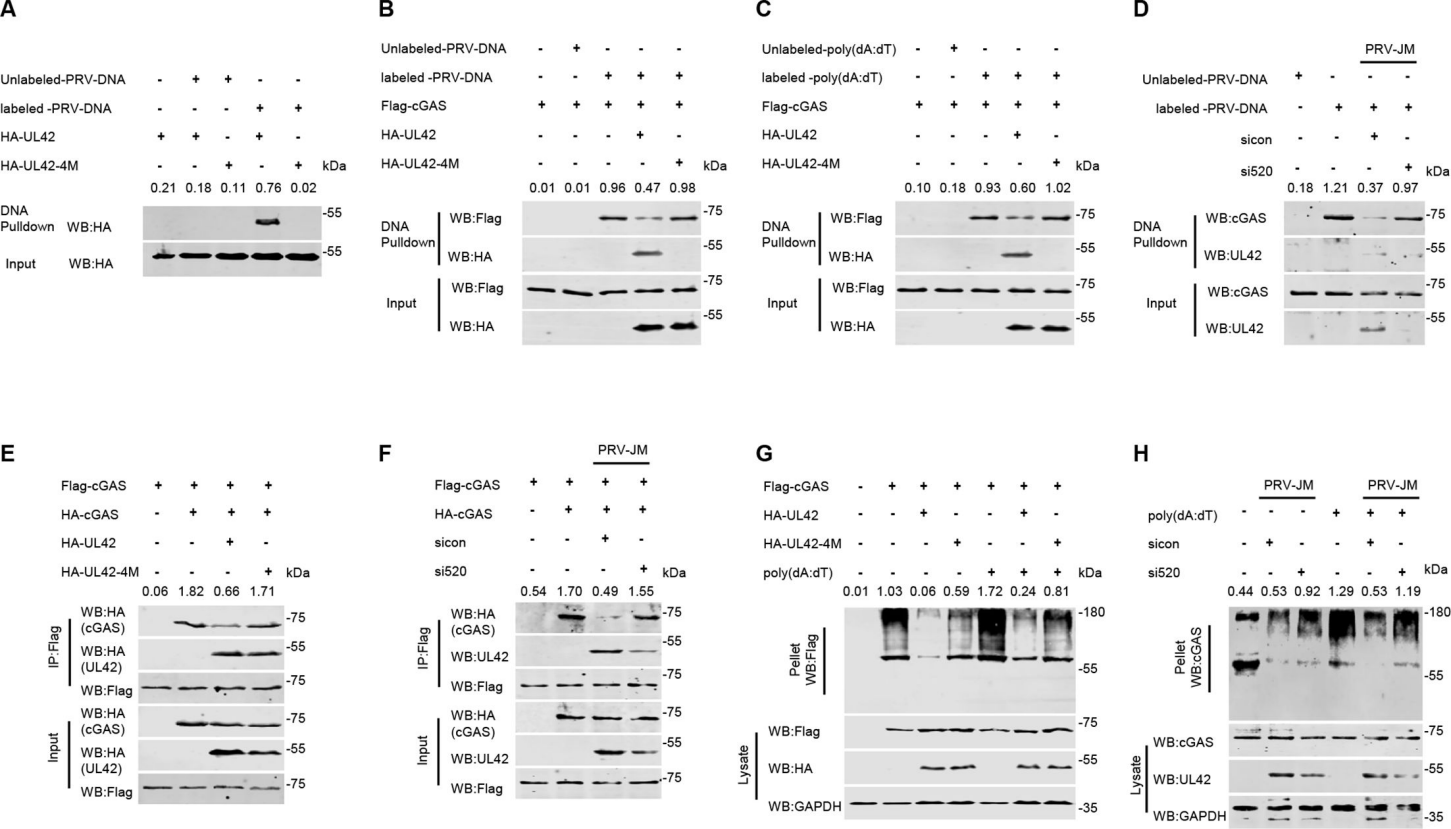
PRV pUL42 inhibits cGAS recognition of dsDNA and oligomerization. (**A**) The effect of pUL42 DNA-binding sites on the recognition of PRV genomic dsDNA. HEK293T cells were transfected with HA-pUL42 or HA-pUL42-4M (2 µg/each) for 24 h. The cell lysates were incubated with biotinylated-PRV genomic dsDNA or unbiotinylated-PRV genomic dsDNA and streptavidin-Sepharose beads for *in vitro* pull-down assays. The bound proteins were then analyzed by immunoblotting with anti-HA. The ratio of the intensity value of the HA-pUL42 immunoblotting result was quantified by ImageJ. (**B**) The effect of pUL42 on the cGAS recognition of PRV genomic dsDNA. HEK293T cells were transfected with Flag-cGAS (2 µg) alone or together with HA-pUL42 or HA-pUL42-4M (2 µg/each) for 24 h. The cell lysates were incubated with biotinylated-PRV genomic dsDNA or unbiotinylated-PRV genomic dsDNA and streptavidin-Sepharose beads for *in vitro* pull-down assays. The bound proteins were then analyzed by immunoblotting with anti-Flag or anti-HA. The ratio of the intensity value of the Flag-cGAS immunoblotting result was quantified by ImageJ. (**C**) The effect of pUL42 on the cGAS recognition of poly(dA:dT). HEK293T cells were transfected with Flag-cGAS (2 µg) alone or together with HA-pUL42 or HA-pUL42-4M (2 µg/each) for 24 h. The cell lysates were incubated with biotinylated-poly(dA:dT) or unbiotinylated-poly(dA:dT) and streptavidin-Sepharose beads for *in vitro* pull-down assays. The bound proteins were then analyzed by immunoblotting with anti-Flag or anti-HA. The ratio of the intensity value of the Flag-cGAS immunoblotting result was quantified by ImageJ. (**D**) The effect of pUL42 knockdown on cGAS recognition of PRV genomic dsDNA. HeLa cells were transfected with si520 or sicon (100 nM/each) for 24 h, and then HeLa cells were infected with PRV (1 MOI) for 24 h. The cell lysates were incubated with biotinylated-PRV genomic dsDNA or unbiotinylated-PRV genomic dsDNA and streptavidin-Sepharose beads for *in vitro* pull-down assays. The bound proteins were then analyzed by immunoblotting with anti-cGAS or anti-pUL42. The ratio of the intensity value of the cGAS immunoblotting result was quantified by ImageJ. (**E**) The effect of pUL42 on the dimerization of cGAS in HeLa cells. HeLa cells were transfected with plasmids expressing Flag-cGAS and HA-cGAS (1 µg/each), together with HA-pUL42 or HA-pUL42-4M (2 µg/each). Co-IP analysis of the interactions of Flag-cGAS and HA-cGAS in the absence or presence of HA-pUL42 or HA-pUL42-4M. The ratio of the intensity value of the HA-cGAS immunoblotting result was quantified by ImageJ. (**F**) The effect of pUL42 knockdown on the dimerization of cGAS in HeLa cells. HeLa cells were transfected with plasmids expressing Flag-cGAS and HA-cGAS (1 µg/each). Subsequently, the cells were transfected with si520 or sicon (100 nM/each) for 24 h, and then HeLa cells were infected with PRV (1 MOI) for 24 h. Co-IP analysis of the interactions of Flag-cGAS and HA-cGAS. The ratio of the intensity value of the HA-cGAS immunoblotting result was quantified by ImageJ. (**G**) The effect of pUL42 on the oligomerization of cGAS in HeLa cells treated with or without poly(dA:dT). HeLa cells were transfected with 2 µg of a plasmid expressing Flag-cGAS alone, or together with the plasmid expressing HA-pUL42 or HA-pUL42-4M (2 µg/each) for 24 h, and then the cells were untreated or treated with 1 µg/mL of poly(dA:dT) for 12 h. The oligomerization of cGAS and the expression of Flag-cGAS, HA-pUL42, HA-pUL42-4M, and GAPDH were detected by Western blotting. The ratio of the intensity value of the Flag-cGAS immunoblotting result was quantified by ImageJ. (**H**) The effect of pUL42 knockdown on the oligomerization of cGAS in HeLa cells induced by PRV. HeLa cells were transfected with si520 or sicon (100 nM/each) for 24 h. HeLa cells were infected with PRV (1 MOI) for 24 h, and then the cells were untreated or treated with 1 µg/mL of poly(dA:dT) for 12 h. The oligomerization of cGAS, and the expression of cGAS, pUL42, and GAPDH were detected by Western blotting. The ratio of the intensity value of the cGAS immunoblotting result was quantified by ImageJ.

The recognition of DNA by cGAS is the first step in activating the cGAS-STING-TBK1 cascade signal. To test whether pUL42 interacts with its DNA-binding region of cGAS to inhibit its recognition of dsDNA, DNA pull-down analysis was performed to measure whether pUL42 has the ability to affect the dsDNA recognition of cGAS. Biotin-labeled PRV genomic DNA was added to HeLa cell lysate expressing Flag-cGAS, HA-pUL42, or HA-pUL42-4M, and a pull-down assay was performed with streptavidin beads. As shown in Fig. 8A, HA-UL42-4M has lost the ability to bind to the PRV genomic DNA. We also found that Flag-cGAS was pulled down by biotin-labeled PRV genomic DNA, but not by biotin-unlabeled PRV genomic DNA. PRV pUL42 significantly inhibited the binding of cGAS to PRV genomic DNA, while pUL42-4M did not (Fig. 8B). Consistent with these results, we also found that Flag-cGAS was pulled down by biotin-labeled poly(dA:dT), but not by biotin-unlabeled poly(dA:dT). pUL42 significantly inhibited the binding of cGAS to poly(dA:dT), while pUL42-4M exhibited a complete loss of this inhibitory capacity (Fig. 8C). To further confirm these results, HeLa cells were transfected with sicon and si520 for 24 h and then infected with PRV-JM (1 MOI) for 24 h. As shown in Fig. 8D, endogenous cGAS can recognize biotin-labeled PRV genomic DNA, while PRV infection inhibits the binding of cGAS to biotin-labeled PRV genomic DNA. Knocking down pUL42 expression completely lost the ability to inhibit the binding of cGAS to PRV genomic DNA during PRV infection in HeLa cells. These data indicate that PRV pUL42 binds to cGAS specifically to prevent the association of cGAS with DNA.

### PRV pUL42 inhibits cGAS oligomerization

cGAS oligomerization is the hallmark of cGAS activation, which is a key step for producing cGAMP (36). Therefore, we analyzed whether PRV pUL42 is required for the inhibition of the dimerization and oligomerization of cGAS. As shown in Fig. 8E, overexpressed pUL42 inhibited the dimerization of cGAS, but pUL42-4M did not. To further confirm this result, HeLa cells were transfected with Flag-cGAS and HA-cGAS for 12 h and then transfected sicon and si520 for 24 h, finally infected with PRV-JM (1 MOI) for 24 h. We also found that PRV infection significantly inhibits the dimerization of cGAS, while knocking down pUL42 completely lost the ability to inhibit the dimerization of cGAS during PRV infection in HeLa cells (Fig. 8F). Consistent with these results, we found that overexpressed pUL42 inhibited the oligomerization of cGAS induced by poly(dA:dT), while pUL42-4M lost the ability to inhibit the oligomerization of cGAS (Fig. 8G). Interestingly, we also found that the oligomerization of cGAS induced by poly(dA:dT) was significantly inhibited during PRV infection in HeLa cells, while knocking down pUL42 lost the ability to inhibit the oligomerization of cGAS during PRV infection in HeLa cells (Fig. 8H). Taken together, these results demonstrated that pUL42 inhibits the dimerization and oligomerization of cGAS during PRV infection.

### PRV pUL42 targets the cGAS-mediated signaling pathway to promote PRV replication

**Fig 9:**
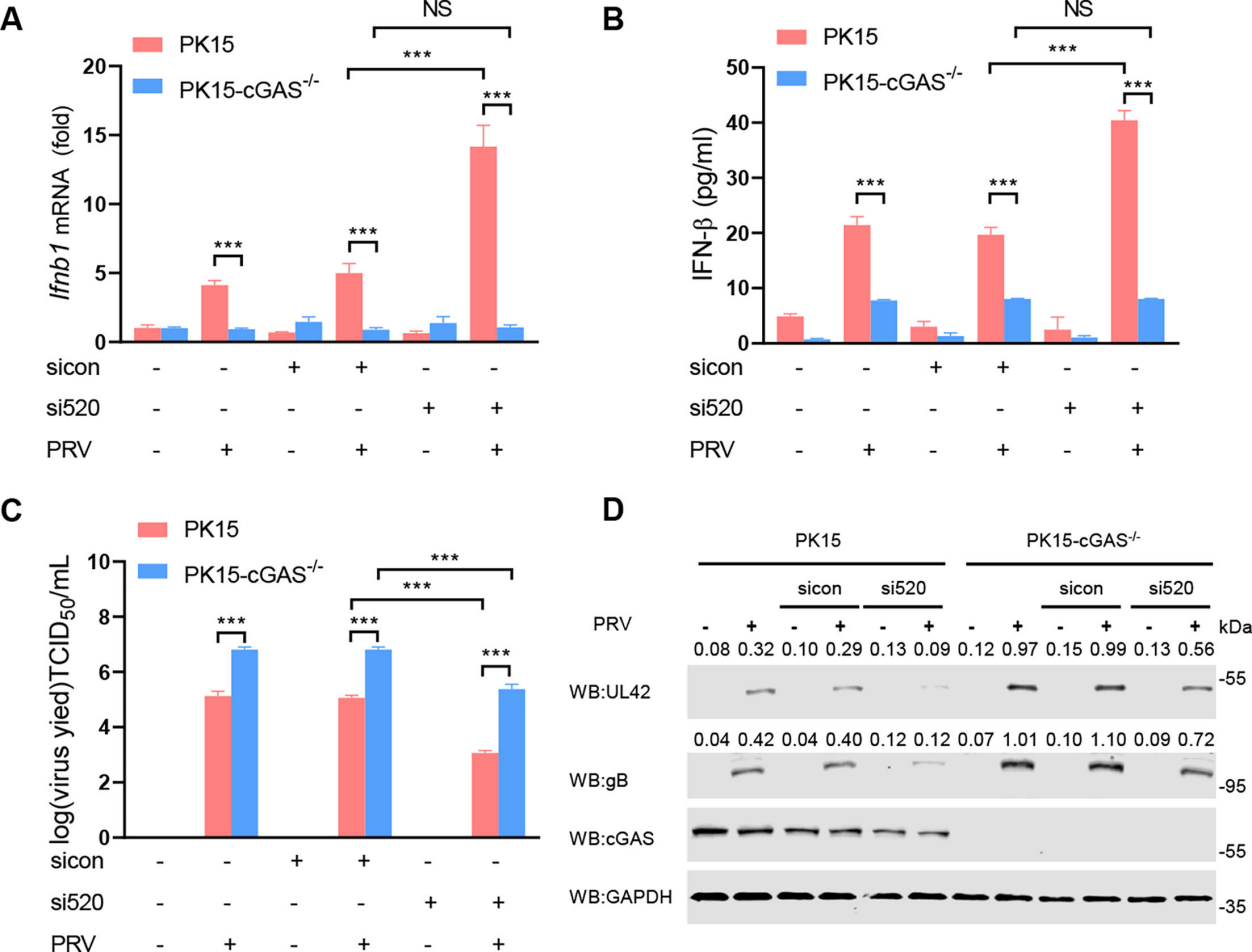
PRV pUL42 targets the cGAS-mediated signaling pathway to promote PRV replication. (**A–D**) Analysis of the production of type І IFN and the viral replication in PK15-WT and PK15-cGAS^-/-^ cells infected with PRV-WT or *Ul42*-knockdown PRV. PK15-WT and PK15-cGAS^-/-^ cells were transfected with si520 or sicon (100 nM/each) for 48 h, then PK15-WT and PK15-cGAS^-/-^ cells were infected with PRV (1 MOI) for 24 h. The mRNA levels of *Ifnb1* (**A**) in cells were detected by qPCR, and the levels of secreted IFN-β in cell supernatants were detected by ELISA (**B**). The titers of PRV-WT or *Ul42*-knockdown PRV infectious progeny virions in PK15-WT and PK15-cGAS^-/-^ cells were detected (**C**). The pUL42, gB, cGAS, and GAPDH proteins of PRV-WT or *Ul42*-knockdown PRV in PK15-WT and PK15-cGAS^-/-^ cells were detected by Western blotting (**D**). The ratio of the intensity value of the pUL42/GAPDH, gB/GAPDH immunoblotting result was quantified by ImageJ. NS, not significant (*P* > 0.05), ***P < 0.001 (two-tailed Student’s t-test).

To examine the inhibitory influence of pUL42 on the cGAS-STING signaling pathway and to assess its implications for efficient viral replication, we conducted the transfection of either sicon or si520 into WT (PK15-WT) or cGAS knockout PK15 (PK15-cGAS^-/-^) cells, followed by infection with PRV to assess the production of type І IFN and the viral replication. In PK15-WT cells, the knockout of cGAS markedly reduces both the mRNA levels of *Ifnb1* and its overall production (Fig. 9A and B), thereby indicating that cGAS is a critical component in the type І IFN production pathway activated by PRV. Additionally, we also found that knocking down *Ul42* significantly promotes the mRNA levels of *Ifnb1* and the production of type І IFN in PK15-WT during PRV infection. However, in PK15-cGAS^-/-^ cells, knocking down *Ul42* has no effect on the production of type І IFN induced by PRV (Fig. 9A and B). This suggests that pUL42 targets the cGAS-mediated signaling pathway to inhibit type І IFN production.

Subsequently, we found that in PK15-WT cells, si520 resulted in a reduction of PRV titer by approximately 2 logs, which reflects the overall performance of pUL42 in viral replication and its function in inhibiting type І IFN production. However, in PK15-cGAS^-/-^ cells, si520 resulted in a decrease in PRV titer by about 1 log, which serves as an evaluation of pUL42’s role in viral replication, particularly given that the knockout of cGAS nearly eliminated type І IFN signaling (Fig. 9C). At the same time, we also observed that the protein levels of pUL42 and gB in PK15-cGAS^-/-^ cells were significantly higher than those in PK15-WT cells (Fig. 9D). In summary, we found that pUL42 targets the cGAS-mediated signaling pathway to enhance viral replication, which is equally significant to pUL42’s role in the viral replication process.

## DISCUSSION

**Fig 10:**
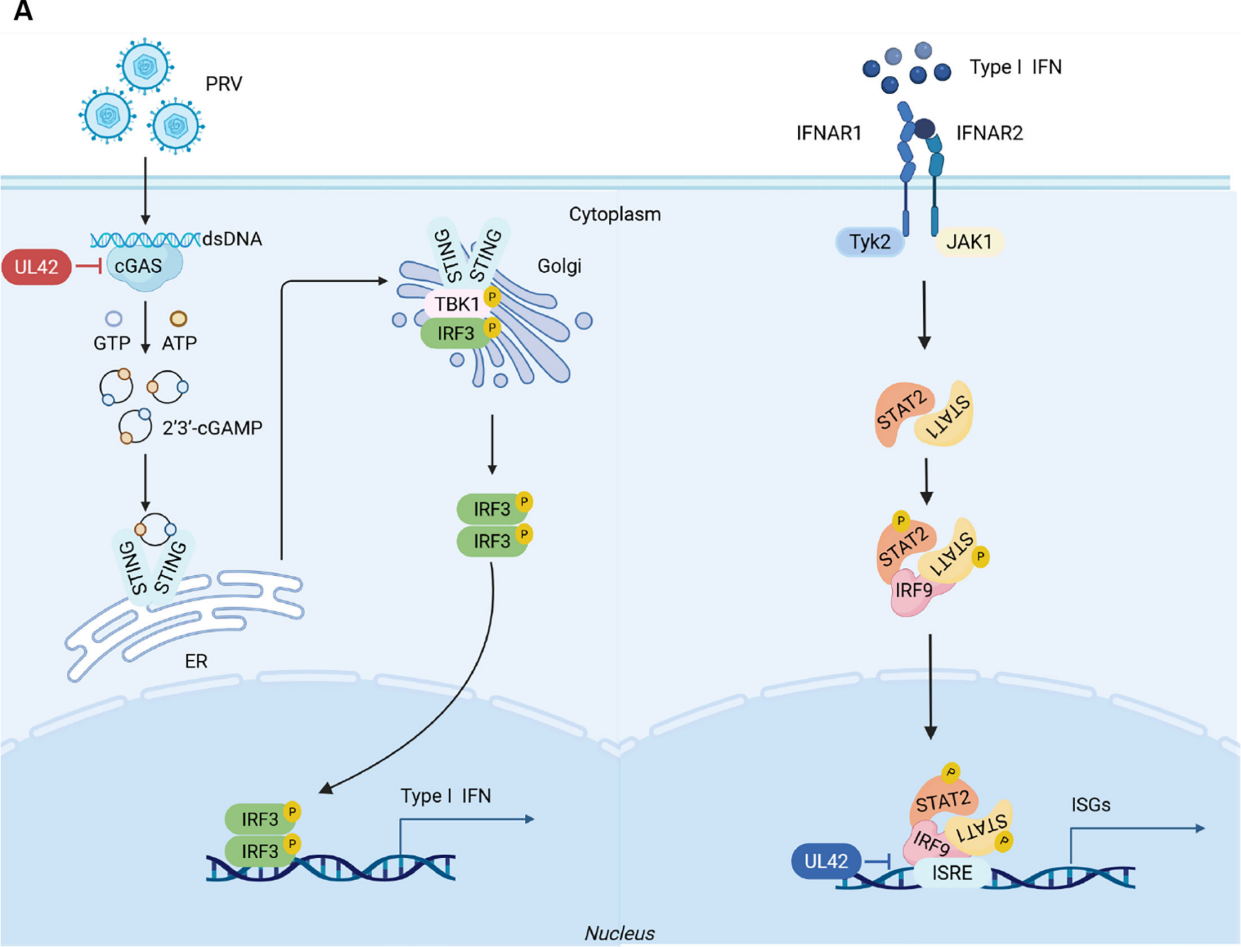
Schematic model of the production of type I IFN inhibited by the PRV pUL42. (**A**) Schematic model of the production of type I IFN inhibited by the PRV pUL42. Upon PRV infection, PRV genomic DNA activates the cGAS-STING signaling pathway and induces type I IFN production. However, PRV pUL42 interacts with cGAS and antagonizes cGAS recognition of dsDNA and its dimerization and oligomerization activation, thereby inhibiting the production of type I IFN. Created in BioRender. Weng, C. (2025) https://BioRender.com/rwztwd9.

cGAS-STING axis is the core pathway for host antiviral immune responses during viral infection. Previous studies have shown that PRVs have evolved multiple strategies to counteract host antiviral responses (1, 37, 38). How PRV targets the cGAS-STING signaling pathway to evade host antiviral response is worth further investigation. In this study, we found that PRV pUL42 is a negative regulator of type I IFN by targeting cGAS. PRV pUL42 interacts with the DNA-binding domain of cGAS and inhibits its recognition of dsDNA during PRV infection, thereby suppressing the oligomerization of cGAS, leading to inhibiting the production of cGAMP and type I IFN. Using siRNA to knock down the expression of the *Ul42* gene, we also revealed that PRV pUL42 is beneficial for PRV replication and evades host antiviral responses by antagonizing the cGAS-mediated innate antiviral immune responses (Fig. 10A).

Upon DNA virus infection, PAMPs in virus-infected cells are sensed by PRRs, such as cGAS, to induce the production of type I IFN (39). The secreted type I IFN binds to IFNAR1/2 on the cell membrane to induce the expression of hundreds of ISGs and exert their antiviral function (40). In this study, we found that PRV infection inhibits the production of type I IFN to promote PRV infection. However, PRV has the ability to antagonize the host’s antiviral immune response, which is crucial for its replication and maintenance of infection in *vivo*. Recently, researchers have shown that several PRV proteins can negatively regulate the production of type I IFN. For example, PRV pUL24 interacts with IFN regulatory factor 7 (IRF7) through the proteasome pathway and degrades IRF7, significantly reducing the production of type I IFN (24). PRV pUL13 recruits E3 ligase RING finger protein 5 (RNF5) to promote K27-/K29-linked ubiquitination of STING, which promotes the degradation of STING, inhibiting type I IFN production (37). Peroxidase 1 (PRDX1) binds to TBK1 and IκB kinase ε (IKKε), actively regulating the production of type I IFN. PRV pUL13 interacts with the antiviral regulatory factor PRDX1 through the ubiquitin proteasome pathway and promotes its degradation, thereby inhibiting-mediated antiviral immune response (41). All these PRV proteins inhibit the production of type I IFN by targeting the downstream components of the cGAS-STING signaling pathway. We proposed that certain PRV proteins may be responsible for targeting cGAS, thereby obstructing the interaction between cGAS and viral DNA. This mechanism is believed to inhibit type I IFN production at an upstream point in the cGAS-STING pathway. Based on unbiased screening, we found that PRV pUL42 is capable of inhibiting the interaction between cGAS and viral genomes, as well as the interaction between cGAS and poly(dA:dT) during PRV infection.

cGAS is activated upon binding to dsDNA, which triggers the synthesis of the second messenger cGAMP. This molecule subsequently activates the STING, thereby initiating an antiviral immune response. HSV-1 possesses mechanisms to inhibit the enzymatic activity of cGAS. Specifically, the HSV-1 pVP22 can directly interact with cGAS, leading to a suppression of its enzymatic function and consequently inhibiting the production of type I IFN (42). Additionally, HSV-1 pUL37 can impede cGAS activation through its deamidase activity, resulting in a reduction of cGAMP synthesis and subsequent blockage of downstream signaling pathways (43). Recent findings have also demonstrated that HSV-1 pUL41 significantly decreases the accumulation of cGAS mRNA, thereby inhibiting the activation of the cGAS-STING-mediated type I IFN signaling pathway (44). Both HSV-1 and PRV pUL21 have been shown to degrade cGAS, facilitating viral infection (45). Furthermore, the envelope protein ORF9 of varicella-zoster virus acts as an antagonist to cGAS, by binding to DNA and phase-separated together with DNA, which may disrupt the cGAS-DNA oligomer and inhibit the activation of the cGAS-STING signaling pathway (46). Our research indicates that PRV pUL42 inhibits the binding of cGAS to dsDNA, thereby suppressing type I IFN production and promoting PRV replication. These findings illustrate that herpesviruses employ various strategies to target cGAS, effectively inhibiting type I IFN production and enhancing viral replication.

DNA sensor cGAS recognizes and binds to viral dsDNA in the cytoplasm to catalyze the production of cGAMP from the substrates GTP and ATP (47). The cGAMP then binds and activates STING to activate host antiviral responses by mediating the production of type I IFN (48). The binding of cGAS to dsDNA forms a 2:2 complex, which is crucial for the activation of cGAS (36, 49). Previous studies have shown that nucleosomes can bind to cGAS and inhibit dsDNA-mediated cGAS activation. The interaction between nucleosomes and cGAS blocks the recognition and binding between cGAS and DNA, disrupting cGAS dimerization and thus “hijacking” it in an inactive monomeric state (50). However, previous studies have shown that cGAS does not have sequence specificity for DNA recognition. In our study, we confirmed that PRV pUL42 interacts with the DNA-binding domain of cGAS and inhibits the dimerization and oligomerization activation of cGAS in *vitro* and in *vivo*.

Alpha herpesvirus pUL42 is a highly conserved DNA polymerase that is an important processing factor for viral DNA replication (18). Functional analysis of HSV-1 pUL42 revealed three main biochemical functions, including binding to DNA, stable binding to viral DNA polymerase pUL30, and acting on increasing the length of DNA chains synthesized by pUL30 (18). Previous studies have shown that the PRV pUL42 can enhance the catalytic activity of DNA polymerase, which is crucial for virus replication. Consistently, we found that knocking down the expression level of the *Ul42* gene significantly inhibited PRV replication, inducing higher levels of type I IFN in PRV-infected cells. It is well known that both PRV pUL42 inhibit the IFN signaling pathway by suppressing the interaction of ISRE-ISGF3 (21). Consistently, we also found that ectopically expressed pUL42 significantly decreased the mRNA levels of *Isg56* and *Isg54* induced by cGAS-STING or by poly(dA:dT), suggesting that PRV pUL42 is a potent inhibitor of both type I IFN production and type I IFN signaling pathway.

Taken together, our findings indicate that the *Ul42* gene has a critical role in PRV replication, and pUL42 can also facilitate PRV replication by inhibiting type I IFN production and the IFN signaling pathway. Even though pUL42 has been shown to inhibit the JAK-STAT signaling pathway induced by IFN-α (21), cGAS functions as an upstream component in the cGAS-STING signaling pathway. So, the inhibition of cGAS-STING signaling through the disruption of the interaction between dsDNA and cGAS by pUL42 is important in the process of PRV infection. Our findings contribute to understanding the function of PRV pUL42 and its role in viral infection, providing new clues to designing anti-PRV or attenuated live vaccines by targeting the PRV *Ul42* gene.

## MATERIALS AND METHODS

### Cells and viruses

PK15 cells, HEK293T cells, and HeLa cells were cultured in Dulbecco’s modified Eagle’s medium (DMEM). PK15-cGAS^-/-^ cells from Professor Beibei Chu of Henan Agricultural University (51). PAMs were isolated from the lung lavage fluid of 4-week-old healthy specific pathogen-free piglets (without African swine fever virus, Classical swine fever virus, Porcine reproductive and respiratory syndrome virus, PRV, and other 28 pathogens), and they were cultured in RPMI 1640 supplemented with 10% fetal bovine serum (FBS), 100 U penicillin, and 100 µg/mL streptomycin at 37°C with 5% CO_2_. PRV-JM was isolated from the aborted piglet samples of PRV-positive pig farms at Jinmen, Guangdong Province of China, as previously described (52).

### Reagents, plasmids, and antibodies

poly(dA:dT) (P0883-10UN) and anti-Flag (M2) beads (M8823) were purchased from Sigma-Aldrich (St. Louis, MO, USA). Protease inhibitor cocktail (4693132001) was purchased from Roche (Basel, Switzerland). DMEM (C11995500CP) and FBS (10091-148) were purchased from GIBCO (Grand Island, NE, USA). The Dual-luciferase Reporter Assay System (E1910) was purchased from Promega (Madison, MI, USA). PrimeScript RT Reagent Kit (RR037A) and SYBR Premix Ex Taq II (RR820A) were purchased from Takara (Shiga, Japan). Rabbit anti-Flag (F7425-2MG) (final dilution 1:1,000), mouse anti-Flag (F1804-1MG) (final dilution 1:1,000), rabbit anti-HA (SAB4300603) (final dilution 1:1,000), and mouse anti-HA (B9183) (final dilution 1:2,000) were purchased from Sigma-Aldrich (St. Louis, MO, USA). Mouse anti-GAPDH (60004-1-Ig) (final dilution 1:10,000), rabbit anti-Lamin B (12987-1-AP) (final dilution 1:1,000), and rabbit anti-TBK1 (28397-1-AP) (final dilution 1:1,000) were purchased from Proteintech (Wuhan, China). Rabbit anti-Phospho-TBK1 (5483) (final dilution 1:1,000), rabbit anti-IRF3 (11904) (final dilution 1:1,000), and rabbit anti-Phospho-IRF3 (29047) (final dilution 1:1,000) were purchased from Cell Signaling Technology (Danvers, MA, USA). Mouse anti-cGAS was from Professor Yong Huang of Northwest A&F University (53). Mouse anti-UL42 was from Professor Liping Huang of Harbin Veterinary Research Institute (54). The IRDye 800CW goat anti-rabbit IgG (H + L) (925-32211) and IRDye 800CW goat anti-mouse IgG (H + L) (925-32210) were purchased from LI-COR (Lincoln, NE, USA). Alexa Fluor 488 goat anti-Rabbit IgG(H + L) (A11008) and Alexa Fluor 594 goat anti-Mouse IgG(H + L) (A11032) were purchased from Thermo Fisher Scientific (Waltham, MA, USA).

The IFN-β-, ISG54-, ISG56-, NF-қB-, ISRE-reporters, and Renilla-TK reporter were obtained from Professor Hong Tang. The 44 cDNAs corresponding to PRV-encoded proteins were synthesized based on the genome of the PRV-JM (52) isolate and cloned into the pCAGGS-Flag (pFlag) vector from GenScript (Nanjing, China). To construct plasmids expressing Flag-tagged or HA-tagged proteins involved in the cGAS-STING signaling pathway, the cDNAs corresponding to these swine genes were amplified by qPCR using total RNA extracted from PAMs as templates and were then cloned into the pCAGGS-Flag or pCAGGS-HA vector, respectively. All constructs were validated by DNA sequencing. The primers used in this study are listed in Table 1.

**TABLE 1:** Primers used for constructing plasmids in this study

Plasmids	Primers (5'−3')
pCAGGS-HA-cGAS	Forward: 5′-ATGGCGGCCCGGCGGGGAAAGTCCACG-3′ Reverse: 5′-GAAATCCATGTCCTCCACCAGGTCCCGGAG-3′
pCAGGS-HA-cGAS-C1	Forward: 5′-ATGGCGGCCCGGCGGGGAAAGTCCACG-3′ Reverse: 5′-GTAGCTCCCGGTGCGCAGCAGGGCAACGC-3′
pCAGGS-HA-cGAS-C2	Forward: 5′-ATGTACTATGAGCGAGTGAAGATTTCTGCTCCCAA-3′ Reverse: 5′-CTTTTCAATGTGAGAGAAGGAAAGCCTCCATGTTT-3′
pCAGGS-HA-cGAS-C3	Forward: 5′-ATGAAGGACATTTTGAAAAATCATGGACAGTCTA-3′ Reverse: 5′-GAAATCCATGTCCTCCACCAGGTCCCGGAG-3′
pCAGGS-HA-STING	Forward: 5′-ATGCCCTACTCCAGCCTGCATCCAT-3′ Reverse: 5′-GAAGATATCTGAGCGGAGTGGAAGAGGCTG-3′
pCAGGS-HA-TBK1	Forward: 5′-ATGCAGAGCACTTCTAATCATCTTTGGCT-3′ Reverse: 5′-AAGACAGTCAACATTGCGAAGGCCACCAT-3′
pCAGGS-HA-IRF3	Forward: 5′-ATGGGAACTCAGAAGCCTCGGATCCTGCC-3′ Reverse: 5′-CGGGGTACCCTAAAGACAGTCAACATTGCGAAGGC-3′
pCAGGS-HA-UL42	Forward:5′-ATGTCGCTGTTCGACGACGGCCTCGAGG-3′ Reverse: 5′-GAATAAATCTCCGTAGGCGTGGC-3′
pCAGGS-HA-UL42-P1	Forward:5′-ATGTCGCTGTTCGACGACGGCCTCGAGG-3′ Reverse:5′-CCGCGGGCGCTTGGCGATGGG-3′
pCAGGS-HA-UL42-P2	Forward:5′-ATGTCGCTGTTCGACGACGGCCTCGAGG-3′ Reverse: 5′-CCCCGCGGCGGGGGAGGCG-3′
pCAGGS-HA-UL42-P3	Forward:5′-ATGAGCGGCGGCGTGCTCGACGCGCT-3′ Reverse: 5′-CCGCGGGCGCTTGGCGATGGG-3′
pCAGGS-HA-UL42-P4	Forward:5′-ATGGTCGACGCGGTCGGCGCGACGGAGCC-3′ Reverse:5′-CCGCGGGCGCTTGGCGATGGG-3′
pCAGGS-HA-UL42-P5	Forward:5′-ATGTCGCTGTTCGACGACGGCCTCGAGG-3′ Reverse:5′-GACGCCCTGGCGCCGCCGGCTGGCGC-3′
pCAGGS-HA-UL42-P6	Forward:5′-ATGTCGCTGTTCGACGACGGCCTCGAGG-3′ Reverse:5′-GCTGCCGTCGACGTTGCCC-3′
pCAGGS-HA-UL42-P7	Forward:5′-ATGAGCGGCGGCGTGCTCGACGCGCTCAAGG-3′ Reverse:5′-GACGCCCTGGCGCCGCCGGCTGGCGC-3′
	

### Cell transfection

HEK293T cells and HeLa cells were transfected with plasmids using PEI or Lipofectamine 2000 transfection reagent. The ratio of plasmid amount to transfection reagent was 1:2 (e.g., 1 µg of plasmid to 2 µL of transfection reagent). Taking one well of a 6-well or 48-well cell culture plate as an example, when the cells grew to 80%–90% confluence, 100 µL of Opti-MEM was added to two sterile 1.5 mL EP tubes, followed by the addition of plasmid and the corresponding transfection reagent to the two EP tubes. The mixtures were gently vortexed to ensure even distribution in Opti-MEM, incubated at room temperature for 5 minutes, and then the Opti-MEM containing the transfection reagent was mixed with the Opti-MEM containing the plasmid. The combined solution was gently vortexed and allowed to sit at room temperature for 20 minutes. Finally, the mixture was slowly added dropwise to the cell culture medium. Samples were collected for subsequent experiments 24–36 hours after transfection.

### Confocal microscopy

HeLa cells were transfected with plasmids expressing HA-tagged or Flag-tagged proteins for 24 h. These cells were fixed with 4% paraformaldehyde and permeabilized with 0.1% Triton X-100. After blocking with 10% FBS, the cells were incubated with anti-Flag and anti-HA antibodies for 2 h. Samples were visualized with a Leica SP2 confocal system (Carl Zeiss AG, Oberkochen, Germany). HeLa cells were infected with PRV (1 MOI) for 0, 4, 8, 12, or 24 h, and the protein level of pUL42 or cGAS was examined with mouse anti-pUL42 or rabbit anti-cGAS antibodies, respectively. The subcellular localization of these proteins was visualized with a Leica SP2 confocal system (Carl Zeiss AG, Oberkochen, Germany).

### Co-immunoprecipitation and Western blot assay

For Co-IP, the cells were lysed in lysis buffer (50 mM Tris-HCl, pH 7.4, 150 mM NaCl, 5 mM MgCl_2_, 1 mM EDTA, 1% Triton X-100, and 10% glycerol) containing 1 mM PMSF and a 1 × protease inhibitor cocktail (Roche, Basel, Switzerland). Then, cell supernatants were incubated with anti-Flag (M2) agarose or with protein G Plus-Agarose immunoprecipitation reagent (Sigma-Aldrich, St. Louis, MO, USA) together with 1 µg of the indicated antibodies at 4°C overnight on a roller. The pellets were washed five times with cell lysis buffer. For Western blot analysis, 20% amounts of cell lysates and immunoprecipitants were resolved by 10%–12% sodium dodecyl sulfate polyacrylamide gel electrophoresis and were then transferred to a polyvinylidene difluoride membrane (Sigma-Aldrich, St. Louis, MO, USA). After incubation with primary and secondary antibodies, the membranes were visualized by enhanced chemiluminescence (Thermo Fisher Scientific, Waltham, MA, USA) or an Odyssey two-color infrared fluorescence imaging system (LI-COR).

### Dual-luciferase reporter assay

HEK293T cells were co-transfected with the indicated plasmids. After 24 h, the cells were harvested and lysed in lysis buffer, and luciferase activities of IFN-β-, ISG54-, ISG56-, NF-қB-, ISRE-reporters, and TK-Renilla reporter were measured with a Dual-luciferase Reporter Assay System (Promega, Madison, MI, USA) according to the manufacturer’s instructions. The data were normalized to the transfection efficiency by dividing the firefly luciferase activity by the Renilla luciferase activity. Each experiment was conducted three times independently, and the representative results are shown.

### Quantitative PCR

To detect the mRNA level *of Ifnb1, Ifnα4, Isg56*, *Isg54,* or *Ul42,* total RNA was extracted using TRIzol reagent (Thermo Fisher Scientific, Waltham, MA, USA), and reverse transcription was performed with a PrimeScript RT Reagent Kit (Takara, Tokyo, Japan). Reverse transcription products were amplified using an Agilent-Strata gene Mx Real-Time qPCR system with SYBR Premix Ex Taq II (Takara, Tokyo, Japan) according to the manufacturer’s instructions. Data were normalized to the level of β-actin expression in individual sample. qPCR was carried out on a QuantStudio5 system (Applied Biosystems, USA) according to the OIE-recommended procedure. All the qPCR primers are listed in Table 2.

**TABLE 2:** Primers used for qPCR in this study

Gene name	Primers	Sequence (5'−3')
Human-IFN-β	h-IFN-β-F h-IFN-β-R	ATGACCAACAAGTGTCTCCTCC GCTCATGGAAAGAGCTGTAGTG
Human-IFN-α	h-IFN-α-F h-IFN-α-R	GAAGAGACTCCCCTGATGAATGT GCACAGGTATACACCAAGCTTCTTC
Human-β-actin	h-β-actin-F h-β-actin-R	CCTTCCTGGGCATGGAGTCCTG GGAGCAATGATCTTGATCTTC
Human-ISG56	h-ISG56-F h-ISG56-R	TTGATGACGATGAAATGCCTGA CAGGTCACCAGACTCCTCAC
Human-ISG54	h-ISG54-F h-ISG54-R	AAGCACCTCAAAGGGCAAAAC TCGGCCCATGTGATAGTAGAC
PRV-UL42	PRV-UL42-F PRV-UL42-R	TGCGTCTACAAGCACGAGTT TAAATCTCCGTAGGCGTGGC
Swine-IFN-β	sq-IFN-β-F	AGCACTGGCTGGAATGAAACCG
	sq-IFN-β-R	CTCCAGGTCATCCATCTGCCCA
Swine-ISG56	sq-ISG56-F	TCAGAGGTGAGAAGGCTGGT
	sq-ISG56-R	GCTTCCTGCAAGTGTCCTTC
Swine-β-actin	sq-β-actin-F	TGAGAACAGCTGCATCCACTT
	sq-β-actin-R	CGAAGGCAGCTCGGAGTT

### RNA interference

The siRNAs targeting the *Ul42* gene are listed in Table 3. The transfection of siRNA was performed with HiPerFect Transfection Reagent (QIAGEN, Germantown, MD) following the manufacturer’s instructions. Forty-eight hours after siRNA transfection, the knockdown efficiency of pUL42 was assessed by Western blotting.

**TABLE 3:** siRNAs targeting STING used in this study

Plasmids	Primers (5'−3')
siNC	5′-UUCUCCGAACGUGUCACGUTT-3′
si385	5′-CGGACCGUCUUCAACGUCATT-3′
si520	5′-CACGAGUUCAACGACUACUTT-3′
si856	5′-GCCGAGCUCACGUUCAACUTT-3′

### DNA pulldown

HEK293T cells were co-transfected with HA-UL42, HA-UL42-4M, or Flag-cGAS for 24 h. Then, the cells were lysed in lysis buffer (50 mM Tris-HCl, pH 7.4, 150 mM NaCl, 5 mM MgCl_2_, 1 mM EDTA, 1% Triton X-100, and 10% glycerol) containing 1 mM PMSF and a 1 × protease inhibitor cocktail (Roche, Basel, Switzerland). Cell supernatants were incubated with biotinylated-PRV-DNA or poly(dA:dT) (Sigma-Aldrich, St. Louis, MO, USA) for 12 h at 4°C, and then incubated with streptavidin beads (Sigma-Aldrich, St. Louis, MO, USA) for another 4  h at 4°C. The beads were washed three times with lysis buffer and analyzed by immunoblotting with the indicated antibodies.

### Statistical analysis

All statistical analyses were performed using one-way ANOVA via the SPSS 16.0 software package (version 16.0, SPSS Inc., Chicago, IL, USA). Data were expressed as the mean ± standard deviation (SD). A value of *P* < 0.05 was considered statistically significant.

## Data Availability

All relevant data are included within the manuscript, and the data supporting the findings of this study are available from the corresponding author upon reasonable request.
